# Supplementary material for: Hydrogen peroxide serves as pivotal fountainhead for aerosol aqueous sulfate formation from a global perspective
Source: Nat Commun. 2024 May 30;15:4625. doi: 10.1038/s41467-024-48793-1 (PMC11139875; doi:10.1038/s41467-024-48793-1)
Supplement: Supplementary file 1 — Supplementary Information [file 41467_2024_48793_MOESM1_ESM.pdf]

Supplementary Information for

Hydrogen peroxide serves as pivotal fountainhead for aerosol aqueous sulfate formation from a global perspective

Jie Gao[1#], Haoqi Wang[1#], Wenqi Liu[1], Han Xu[1], Yuting Wei[1], Xiao Tian[1], Yinchang Feng[1], Shaojie Song[1*] and Guoliang Shi[1*]

[1]State Environmental Protection Key Laboratory of Urban Ambient Air Particulate Matter Pollution Prevention and Control, Tianjin Key Laboratory of Urban Transport Emission Research, China Meteorological Administration-Nankai University Cooperative Laboratory for Atmospheric Environment-Health Research, College of Environmental Science and Engineering, Nankai University, Tianjin 300350, China.

[#] These authors contributed equally: Jie Gao, Haoqi Wang.

[*] Corresponding authors: S. Song (songs@nankai.edu.cn) and G. Shi (nksgl@nankai.edu.cn)

TABLE OF CONTENTS

Number of Texts: 4

Number of Tables: 2

Number of Figures: 25

This document contains the following:

Text S1. Calculations of aqueous-phase concentrations. 3
Table S1. Constants for calculating the aqueous-phase concentrations. 3
Text S2. The aqueous oxidation reaction of S(IV) into SO_4^{2-}. 4
Table S2. Rate expressions and rate constants of relevant aqueous reactions. 4
Text S3. Kinetics of mass transport. 5
Text S4. Improved kinetics of mass transport. 6
Fig. S1. Aerosol sulfate formation by four aqueous pathways at the surface layer in January 2019. 8
Fig. S2. Aerosol sulfate formation by four aqueous pathways at the surface layer in April 2019. 9
Fig. S3. Aerosol sulfate formation by four aqueous pathways at the surface layer in July 2019. 10
Fig. S4. Aerosol sulfate formation by four aqueous pathways at the surface layer in October 2019. 11
Fig. S5. Aerosol ionic strength at the surface layer in 2019. 12
Fig. S6. Aerosol pH at the surface layer in 2019. 13
Fig. S7. SO_4^{2-} and SO_2 concentration at the surface layer in 2019. 14
Fig. S8. Aerosol sulfate formation by OH gas-phase oxidation at the surface layer in 2019. 15
Fig. S9. Aerosol pH and aerosol sulfate formation rates through four aqueous oxidation pathways at different atmospheric pressure levels in January 2019. 16
Fig. S10. Aerosol pH and aerosol sulfate formation rates through four aqueous oxidation pathways at different atmospheric pressure levels in July 2019. 17
Fig. S11. Vertical distribution of aerosol pH, AWC, H_2O_2, NO_2, O_3, Mn(II), and Fe(III), averaged by latitude. 19
Fig. S12. Vertical distribution of SO_2 and SO_4^{2-} averaged by latitude. 20
Fig. S13. The global difference in pH at 950 mbar between January and July 2019. 21
Fig. S14. Aerosol aqueous sulfate formation pathways in ten atmospheric layers near the surface of the typical urban areas. 22
Fig. S15. Drivers of H_2O_2 pathway in typical urban areas in recent 20 years. 23
Fig. S16. Drivers of NO_2 pathway in typical urban areas in recent 20 years. 24
Fig. S17. Drivers of O_3 pathway in typical urban areas in recent 20 years. 25
Fig. S18. Drivers of TMI pathway in typical urban areas in recent 20 years. 26
Fig. S19. Changes in aerosol sulfate production by the H_2O_2 pathway with fixation of H_2O_2, SO_2, pH, and AWC at 2001 levels. 27
Fig. S20. Spatial distribution of $PM_{2.5}$ concentration from GEOS-Chem simulation and satellite-derived reanalysis dataset in January, April, July, and October 2019. 28
Fig. S21. Comparison of $PM_{2.5}$ concentration between GEOS-Chem simulation and satellite-derived reanalysis dataset in North Africa, Oceania, East Asia, Europe, and North America. 29
Fig. S22. $SO_4^{2-}/PM_{2.5}$ from GEOS-Chem simulation and observation in 2019. 30
Fig. S23. Comparison of $SO_4^{2-}/PM_{2.5}$ between GEOS-Chem simulation and observation in East Asia, Europe, and North America. 30
Fig. S24. Aerosol pH from GEOS-Chem simulation and observation-based estimation in January, April, July, and October. 31
Fig. S25. Comparison of aerosol pH between GEOS-Chem simulation and observation-based estimation in East Asia, South Asia, Europe, and North America. 31
Supplementary References 32

Text S1. Calculations of aqueous-phase concentrations.

The dissolution of gas species (SO_2 and oxidants) in the aqueous phase follows Henry's law.

$$[X(aq)]=H_X \times P_{X(g)} \qquad (S1)$$

where [X(aq)] is the aqueous-phase concentration of X species in equilibrium with $P_{X(g)}$, mol L^{-1}; H_X is Henry's law coefficient, mol L^{-1} atm^{-1}; and $P_{X(g)}$ is the partial pressure of X in the gas phase, atm.

Henry's constants and ionization constants are dependent on temperature. The temperature dependence of an equilibrium constant is given by the van't Hoff equation [1].

$$\frac{d\ln H_X}{dT}=\frac{\Delta H_X}{RT^2} \qquad (S2)$$

where ΔH_X is the enthalpy change at constant temperature and pressure; R is the molar gas constant, 8.314 J mol^{-1} K^{-1}; T is the temperature, K. ΔH_X is a function of temperature, but it is approximately constant over small temperature ranges. Thus,

$$H_T=H_{T_0}\exp[-\frac{\Delta H_{298K}}{R}(\frac{1}{T}-\frac{1}{T_0})] \qquad (S3)$$

where T_0 can be 298K; H_{T_0} is the Henry's constant at 298K, mol L^{-1} atm^{-1}. H_T is Henry's constant at a specific temperature. Table. S1 shows more details [1,2].

Table S1. Constants for calculating the aqueous-phase concentrations.

Gas species	Aqueous phase concentrations	H_{298K} (M atm^{-1}) or K_{298K} (M)	$-\triangle H_{298K}/R$ (K)
	$[SO_2\cdot H_2O(aq)]=H_{SO_2}\times p_{SO_2}$	1.23	3145.3
SO_2	$[HSO_3^-(aq)]=K_{s1}\times[SO_2\cdot H_2O(aq)]/[H^+]$	1.3×10^{-2}	1960
	$[SO_3^{2-}(aq)]=K_{s2}\times[HSO_3^-(aq)]/[H^+]$	6.6×10^{-8}	1500
H_2O_2	$[H_2O_2(aq)]=H_{H_2O_2}\times p_{H_2O_2}$	1×10^{5}	7297.1
NO_2	$[NO_2(aq)]=H_{NO_2}\times p_{NO_2}$	1×10^{-2}	2516.2
O_3	$[O_3(aq)]=H_{O_3}\times p_{O_3}$	1.1×10^{-2}	2536.4

Text S2. The aqueous oxidation reaction of S(IV) into SO_4^{2-}.

The aqueous-phase reaction equations of four oxidants are different.

For H_2O_2 oxidation pathway [1],

$H_2O_2 + HSO_3^- \rightleftharpoons SO_2OOH^- + H_2O$

$SO_2OOH^- + H^+ \rightarrow H_2SO_4$

For NO_2 oxidation pathway [3],

$2NO_2 + HSO_3^- + H_2O \rightarrow 2HONO + SO_4^{2-} + H^+$

$2NO_2 + SO_3^{2-} + H_2O \rightarrow 2HONO + SO_4^{2-}$

For O_3 oxidation pathway [1],

$O_3 + SO_2 \cdot H_2O \rightarrow 2H^+ + SO_4^{2-} + O_2$

$O_3 + HSO_3^- \rightarrow H^+ + SO_4^{2-} + O_2$

$O_3 + SO_3^{2-} \rightarrow SO_4^{2-} + O_2$

For O_2 +TMI oxidation pathway [1,4],

$1/2\ O_2 + S(IV) \xrightarrow{Mn^{2+},\ Fe^{3+}} S(VI)$

The corresponding rate expressions and rate constants for each pathway can be referred to Table S2.

Table S2. Rate expressions and rate constants of relevant aqueous reactions.

Oxidant	Reaction rate expressions ($R_{oxidant+S(IV)}$)	Rate constants (k)
H_2O_2	$k_1[H^+][HSO_3^-][H_2O_2(aq)]/(1+K[H^+])$	$k_1 = 7.45\times10^7\times e^{(-4430\times(1/T-1/298))}\ M^{-1}\ s^{-1}$<br>$K = 13\ M^{-1}$
NO_2	$(k_2/[H^+]+K_{s2}k_3/[H^+]^2)K_{s1}[SO_2\cdot H_2O(aq)][NO_2(aq)]$	$k_2 = 10^6\ M^{-1}\ s^{-1}$<br>$k_3 = 10^{10}\ M^{-1}\ s^{-1}$
O_3	$k_4[SO_2\cdot H_2O]+k_5[HSO_3^-]+k_6[SO_3^{2-}])[O_3(aq)]$	$k_4 = 2.4\times10^4\ M^{-1}\ s^{-1}$<br>$k_5 = 3.7\times10^5\times e^{(-5530\times(1/T-1/298))}\ M^{-1}\ s^{-1}$<br>$k_6 = 1.5\times10^9\times e^{(-5280\times(1/T-1/298))}\ M^{-1}\ s^{-1}$
O_2 +TMI	$\begin{cases} k_7[H^+]^{-0.74}[S(IV)][Mn(II)][Fe(III)], & pH \leq 4.2 \\ k_8[H^+]^{0.67}[S(IV)][Mn(II)][Fe(III)], & pH > 4.2 \end{cases}$	$k_7 = 3.72\times10^7\times e^{(-8431.6\times(1/T-1/297))}\ M^{-2}\ s^{-1}$<br>$k_8 = 2.51\times10^{13}\times e^{(-8431.6\times(1/T-1/297))}\ M^{-2}\ s^{-1}$

Text S3. Kinetics of mass transport.

The overall reaction rate for S(IV) oxidation depends on both chemical reaction rates in the aqueous phase and on the mass transport limitations that may exist across the interface and in different mediums for multiphase reactions. The original standard resistance model can evaluate the effects of mass transport [2,5]:

$$\frac{1}{R_{H,\ oxidant+S(IV)}}=\frac{1}{R_{oxidant+S(IV)}}+\frac{1}{J_{aq,lim}} \quad (S4)$$

where $R_{H,\ oxidant+S(IV)}$ is the overall production rate for S(IV) oxidation by a certain oxidant, such as H_2O_2, NO_2, O_3, and O_2+TMI, mol L^{-1} s^{-1}; $R_{oxidant+S(IV)}$ is the aqueous-phase reaction rate, mol L^{-1} s^{-1}; $J_{aq,lim}$ is the rate limited by mass transfer from gas to the aqueous phase, mol L^{-1} s^{-1}, which is determined by:

$$J_{aq,lim}=\min\{J_{aq}(SO_2),\ J_{aq}(\text{oxidant})\} \quad (S5)$$

$$J_{aq}(X)=k_{MT}(X)\times[X(aq)] \quad (S6)$$

where [X(aq)] is the aqueous phase concentration of SO_2 or oxidants, which is determined by Henry's law:

$$[X(aq)]=H_X\times P_{X(g)} \quad (S7)$$

where H_X is Henry's law constant, mol L^{-1} atm^{-1}; $P_{X(g)}$ is the partial pressure of X in the gas phase, atm.

The mass transfer rate coefficient K_{MT} (s^{-1}) accounting for gas-phase diffusion and interfacial transport could be calculated by:

$$k_{MT}(X)=\left[\frac{R_p^2}{3D_g}+\frac{4R_p}{3\alpha v}\right]^{-1} \quad (S8)$$

where $\frac{R_p^2}{3D_g}$ is the continuum regime resistance; $\frac{4R_p}{3\alpha v}$ is the free-molecular (or kinetic) regime resistance. R_p is the radius of aerosol particles (an equivalent R_p of 1.5×10^{-7} m was adopted); D_g is the gas-phase molecular diffusion coefficient (the typical tropospheric value is 2×10^{-5} m^2 s^{-1}); α is the mass accommodation coefficient on droplet surface (0.11 for SO_2, 0.23 for H_2O_2, 2×10^{-3} for O_3, and 2×10^{-4} for NO_2); v is the mean molecular speed (the typical tropospheric value was 300 m s^{-1}).

Text S4. Improved kinetics of mass transport.

Compared with the dilute solution, the high ionic strength of aerosol could influence the salting effects, dissolution of S(IV) into its ionic forms, and the rate constants of chemical reactions, etc., thereby playing a part in the oxidation rate of sulfate formation pathway in the aerosol aqueous phase [6,7]. To consider the effect of ionic strength, the improved standard resistance model was adopted here [2,8]:

$$\frac{1}{R_{H,\ oxidant+S(IV)}} = \frac{1}{EF_{oxidant} \cdot R_{oxidant+S(IV)}} + \frac{1}{J_{aq,lim}} \quad (S9)$$

where $R_{H,\ oxidant+S(IV)}$ is the overall reaction rate for S(IV) oxidation by a certain oxidant, mol L^{-1} s^{-1}; $R_{oxidant+S(IV)}$ is the aqueous-phase reaction rate, mol L^{-1} s^{-1}; $J_{aq,lim}$ is the rate limited by mass transfer from gas phase to aqueous phase, mol L^{-1} s^{-1}; and $EF_{oxidant}$ is the enhancement factor of the corresponding oxidation pathway caused by the high ionic strength. EFs of the reaction rate in aerosols relative to the dilute solution were used to describe the overall effects of the solute strength [6].

Firstly, the recent study has indicated an enhancement factor of 33-51 for the kinetics of H_2O_2 oxidation at an ionic strength of 14 mol kg^{-1}, compared with the rate in the dilute solution; while the formation rate through TMI oxidation was suppressed by a factor of approximately 85 at an ionic strength of 2.8 mol kg^{-1} [9]. Based on previous work [8,9], we adopted conservative EF values here to conduct global simulation, i.e., 30 for the H_2O_2 pathway and 0.8 for the TMI pathway. Moreover, some studies have proposed an enhancement effect of ionic strength on sulfate formation by NO_2 oxidation [2,10]. The recent experimental study reported that the effective rate constant of the reaction between NO_2 and SO_3^{2-} in atmospherically deliquesced particles was more than 3 orders of magnitude larger than that in dilute solution [3]. Whereas the effective rate constant for the reaction of NO_2 with HSO_3^{2-} was accordant with the value obtained in bulk solutions [3]. This study provided new kinetic data for quantitatively understanding the mechanism of NO_2 oxidation of SO_2, which was then applied in our global simulation. Besides, in sea salt aerosols, the essential role of ionic strength in O_3 aqueous oxidation has also been recognized, namely, the formation rate of the O_3 pathway could be enhanced by a factor of 7.9-233 under high ionic strength conditions of 2-14 mol kg^{-1}, compared with the dilute bulk solutions [11]. In this work, we

focus on the continental regions. According to the previous work [8], overall reaction in alkaline aerosols would be limited by mass transfer rate; and overall rates would still be lower than other pathways after adding high EF in acidic aerosols. Therefore, the EF for the O_3 pathway was determined as 1 here. Despite prior studies, deviations and uncertainties of aerosol aqueous oxidations still exist. More knowledge of the kinetics and thermodynamics in the high ionic strength solution is still needed [6,10].

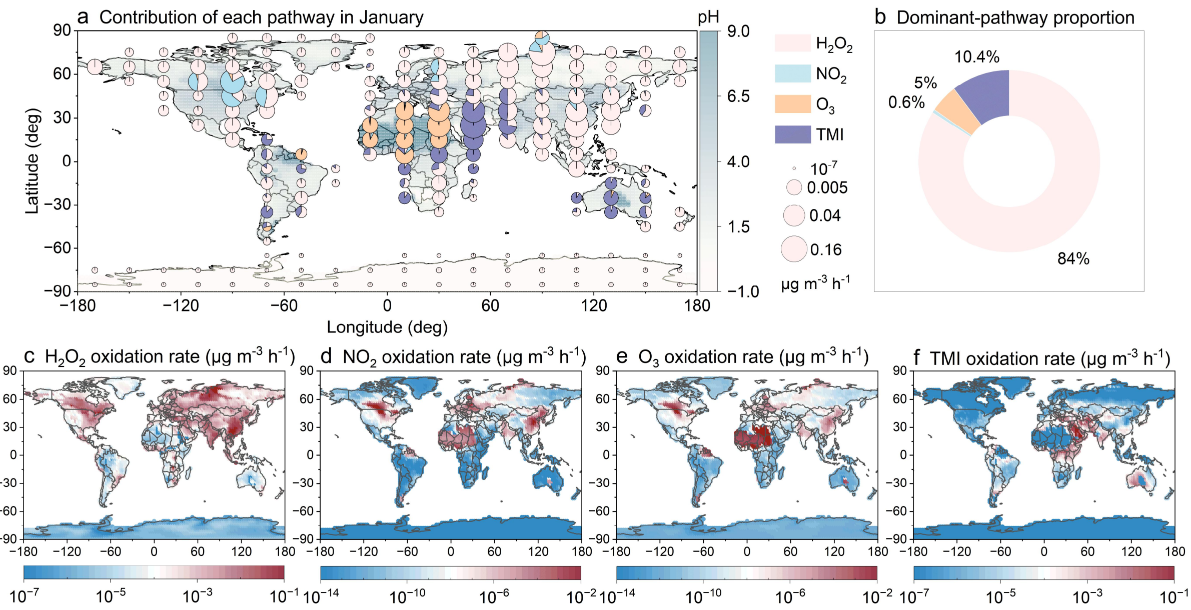

Fig. S1. Aerosol sulfate formation by four aqueous pathways at the surface layer in January 2019.

(a) The distribution of average contribution by each aerosol aqueous oxidation pathway, which was counted for every 20 longitudes and every 10 latitudes. The size of the pie chart represents the total oxidation contribution summed with the four pathways. (b) The proportion dominated by different oxidation routes on the surface in continental areas. (c-f) The distribution of oxidation rate of four pathways. H_2O_2 oxidation showed the dominant effect on global sulfate formation in January. The oxidation rates of the O_3, NO_2, and TMI pathways were mostly lower than that of the H_2O_2 pathway, though they were pivotal in some partial areas.

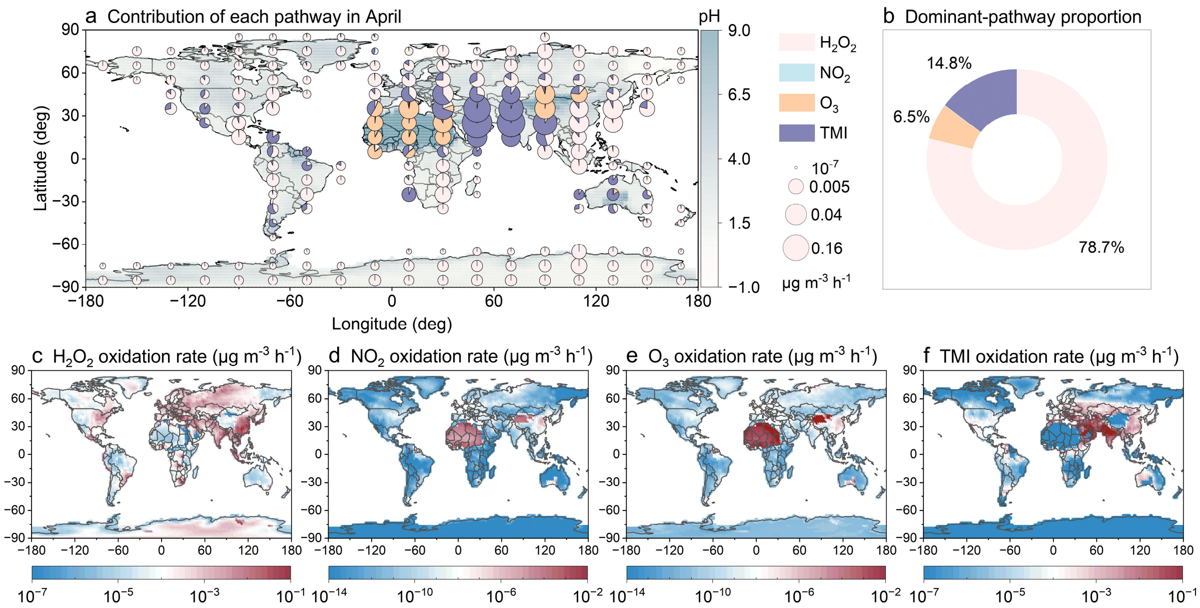

Fig. S2. Aerosol sulfate formation by four aqueous pathways at the surface layer in April 2019.

(a) The distribution of average contribution by each aerosol aqueous oxidation pathway, which was counted for every 20 longitudes and every 10 latitudes. The size of the pie chart represents the total oxidation contribution summed with the four pathways. (b) The proportion dominated by different oxidation routes on the surface in continental areas. (c-f) The distribution of oxidation rate of four pathways. Besides H_2O_2 oxidation, the significant contribution of TMI oxidation increased in April, especially in South Asia and West Asia, resulting in a higher dominant proportion.

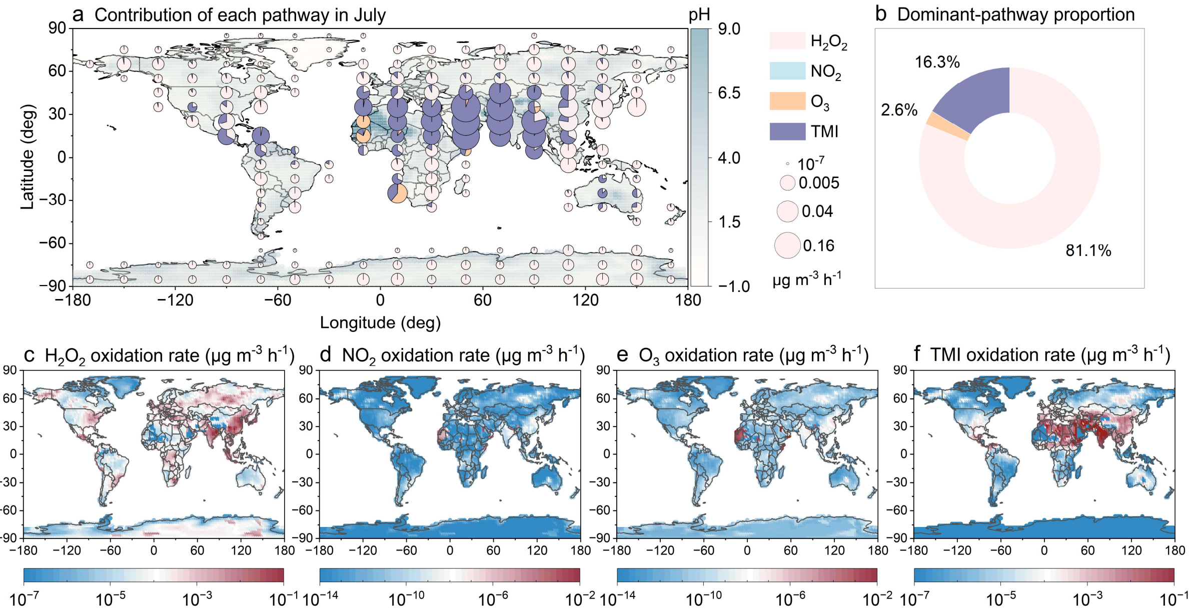

Fig. S3. Aerosol sulfate formation by four aqueous pathways at the surface layer in July 2019.

(a) The distribution of average contribution by each aerosol aqueous oxidation pathway, which was counted for every 20 longitudes and every 10 latitudes. The size of the pie chart represents the total oxidation contribution summed with the four pathways. (b) The proportion dominated by different oxidation routes on the surface in continental areas. (c-f) The distribution of oxidation rate of four pathways. H_2O_2 oxidation was still the main pathway. However, similar to April, the significant role of the TMI pathway became more notable in July. The regions dominated by the TMI pathway expanded, enrolling some areas of North Africa. The contribution by NO_2 and O_3 pathways was relatively low in July.

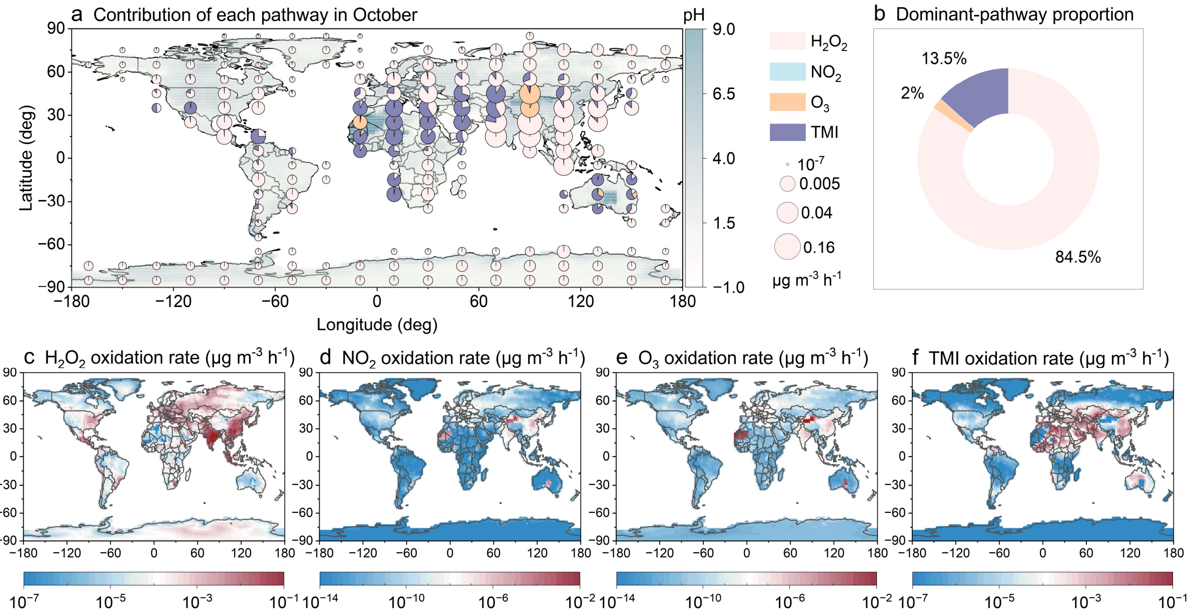

Fig. S4. Aerosol sulfate formation by four aqueous pathways at the surface layer in October 2019.

(a) The distribution of average contribution by each aerosol aqueous oxidation pathway, which was counted for every 20 longitudes and every 10 latitudes. The size of the pie chart represents the total oxidation contribution summed with the four pathways. (b) The proportion dominated by different oxidation routes on the surface in continental areas. (c-f) The distribution of oxidation rate of four pathways. H_2O_2 oxidation was still of the essence in October. TMI pathway would prevail in some areas of North Africa and West Asia. H_2O_2 and TMI pathways had greater oxidation rates than NO_2 and O_3 pathways.

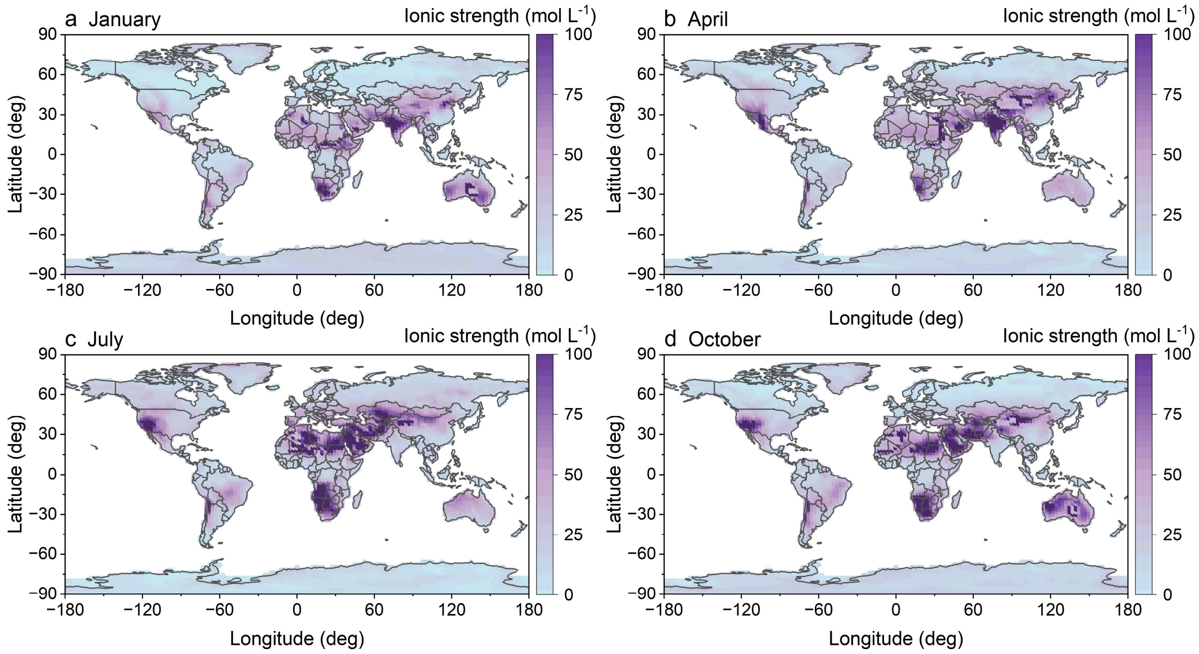

Fig. S5. Aerosol ionic strength at the surface layer in 2019.

Ionic strength in (a) January, (b) April, (c) July, and (d) October exhibits seasonal features.

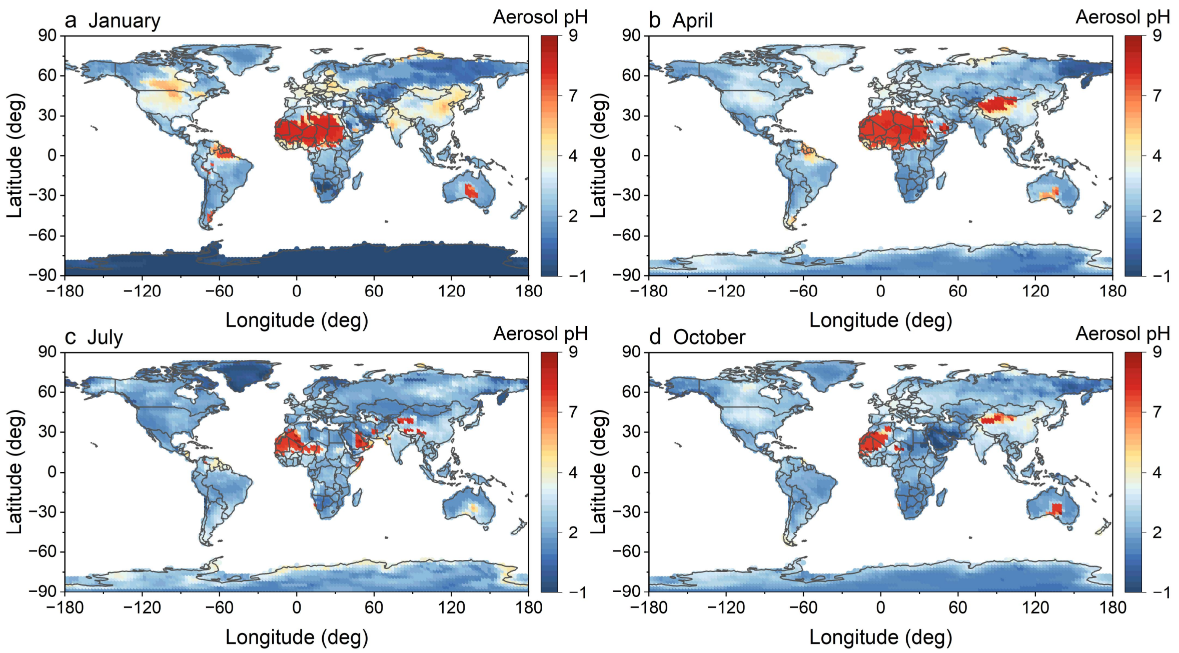

Fig. S6. Aerosol pH at the surface layer in 2019.

Aerosol pH in (a) January, (b) April, (c) July, and (d) October showed that fine-mode aerosol was universally acidic in anthropogenically dominated locations [12], except for desert dust aerosol. Ambient particles in the Sahara Desert were predicted to be alkaline, due to strong influences of nonvolatile cations in this region affected by dust. Seasonal variation of aerosol acidity also showed a similar trend in most locations, namely, the minimum value of aerosol pH would be present in summer [12].

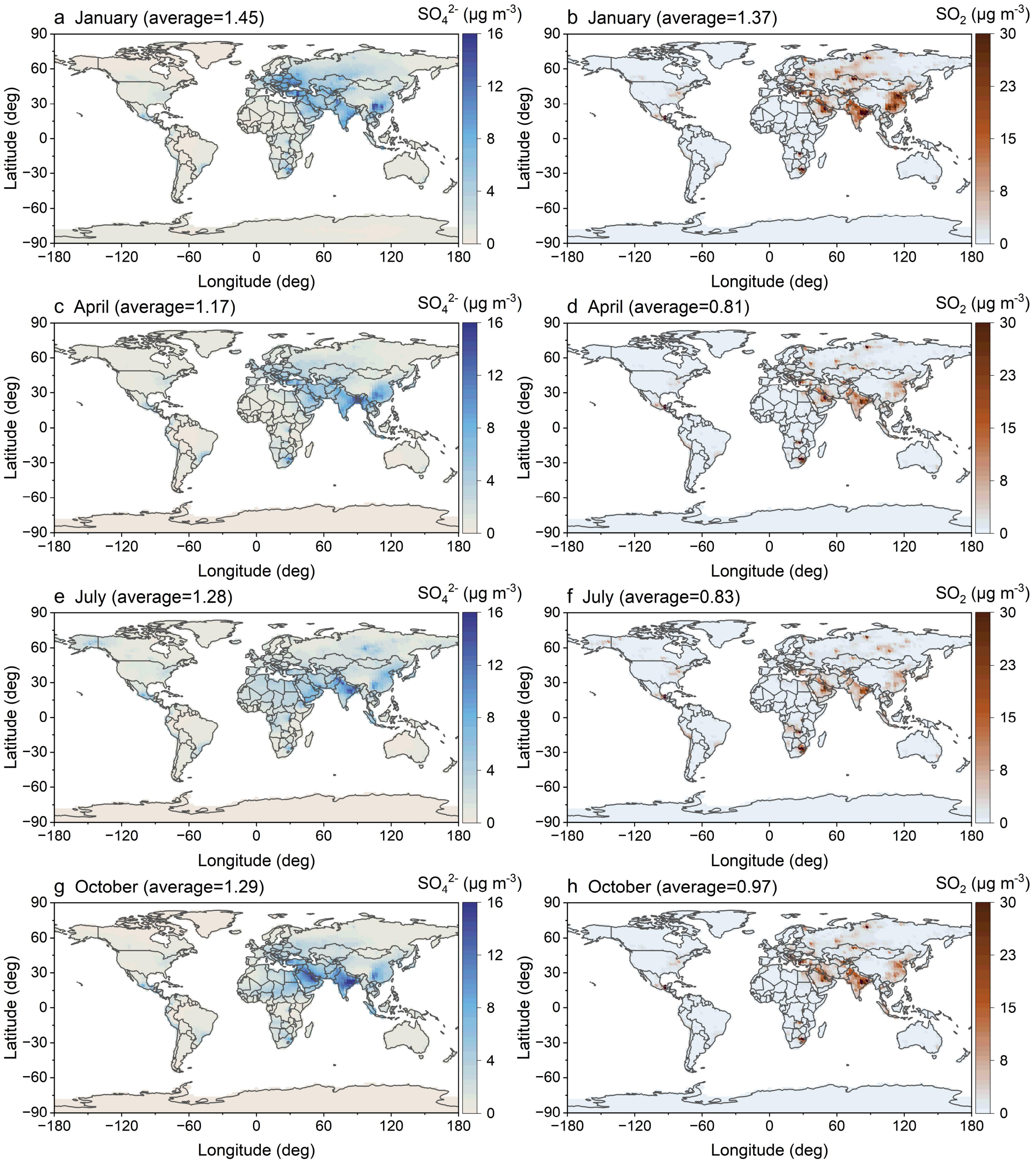

Fig. S7. SO_4^{2-} and SO_2 concentration at the surface layer in 2019.

The spatial distribution of SO_4^{2-} concentration was shown in (a) January, (c) April, (e) July, and (g) October. The spatial distribution of SO_2 concentration was shown in (b) January, (d) April, (f) July, and (h) October. The SO_4^{2-} concentration at the surface layer exhibited a large spatial variability, and corresponded well with the spatial distribution of SO_2, showing higher levels in the northern Hemisphere. In some populated continental regions, SO_4^{2-} and SO_2 were higher in January and October.

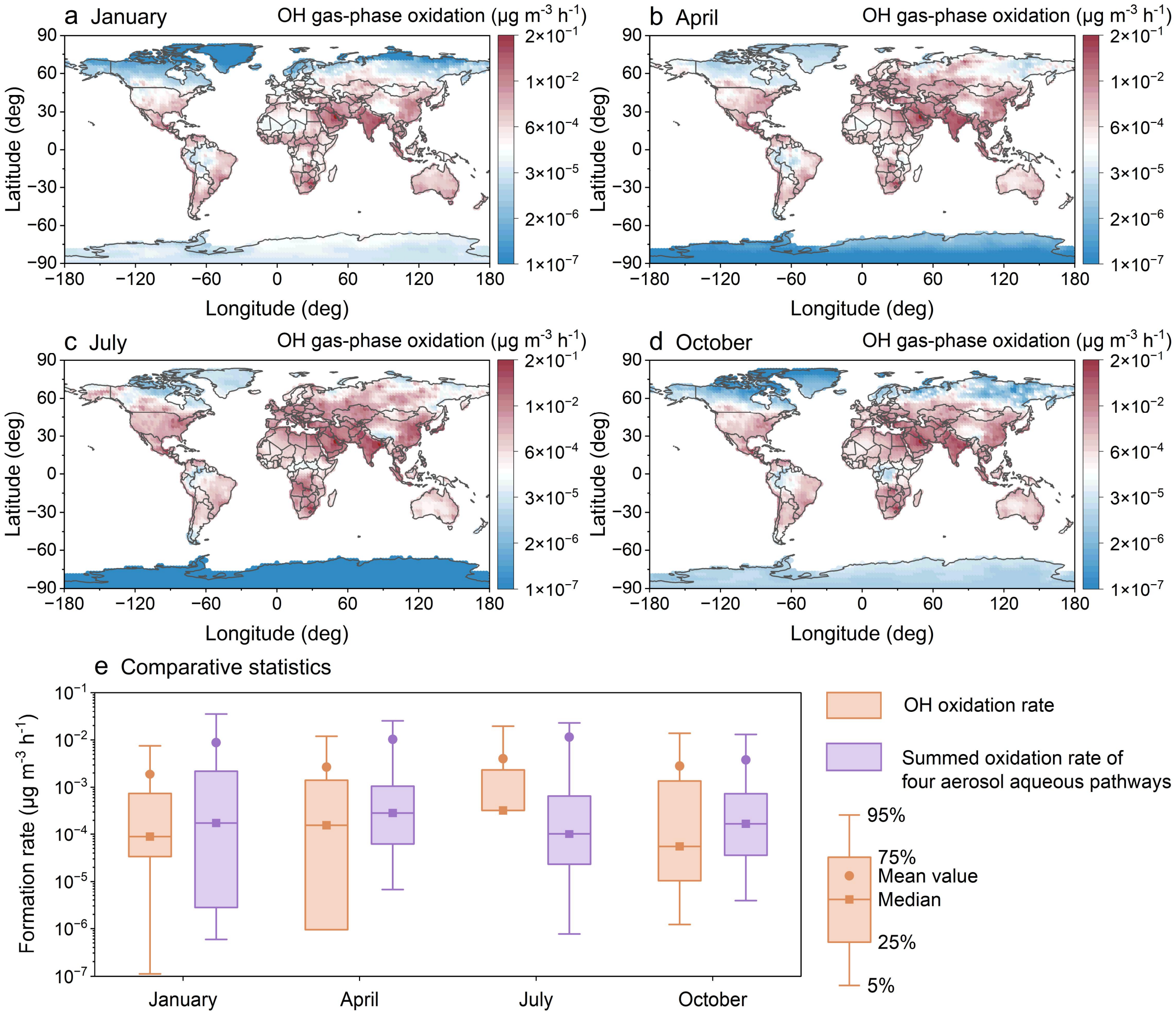

Fig. S8. Aerosol sulfate formation by OH gas-phase oxidation at the surface layer in 2019. The spatial distribution of OH gas-phase oxidation rate was shown in (a) January, (b) April, (c) July, and (d) October. (e) Comparative statistics showed the comparison of sulfate formation rates for OH gas-phase oxidation and summed aerosol aqueous-phase oxidation. The sulfate formation rates from OH gas-phase oxidation based on GEOS-Chem simulations were comparable with the sulfate formation rates of summed aerosol aqueous-phase oxidations based on the combination of the GEOS-Chem simulations and subsequent theoretical calculations.

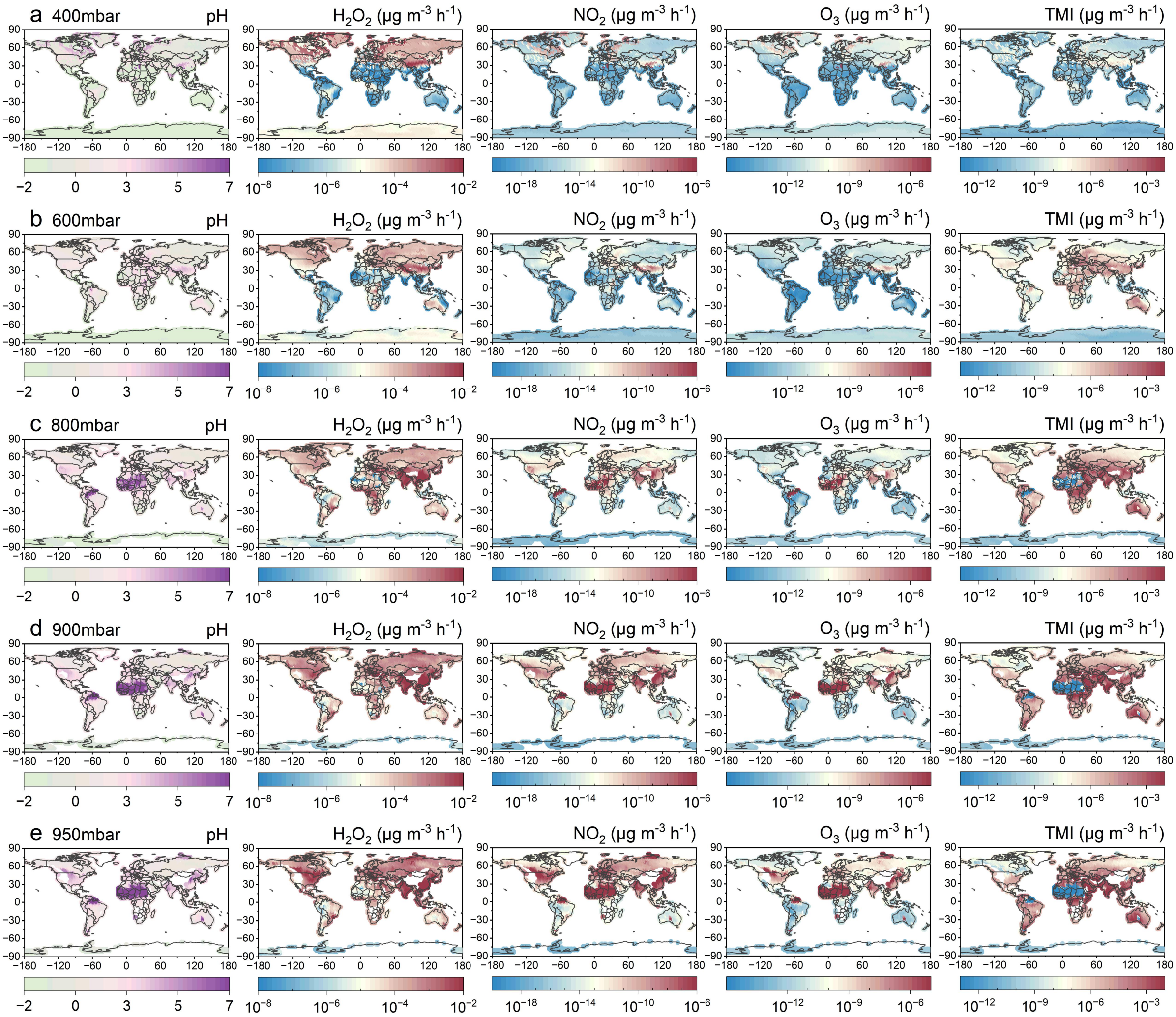

Fig. S9. Aerosol pH and aerosol sulfate formation rates through four aqueous oxidation pathways at different atmospheric pressure levels in January 2019.

(a)-(e) indicated the results at 400 mbar, 600 mbar, 800 mbar, 900 mbar, and 950 mbar, respectively.

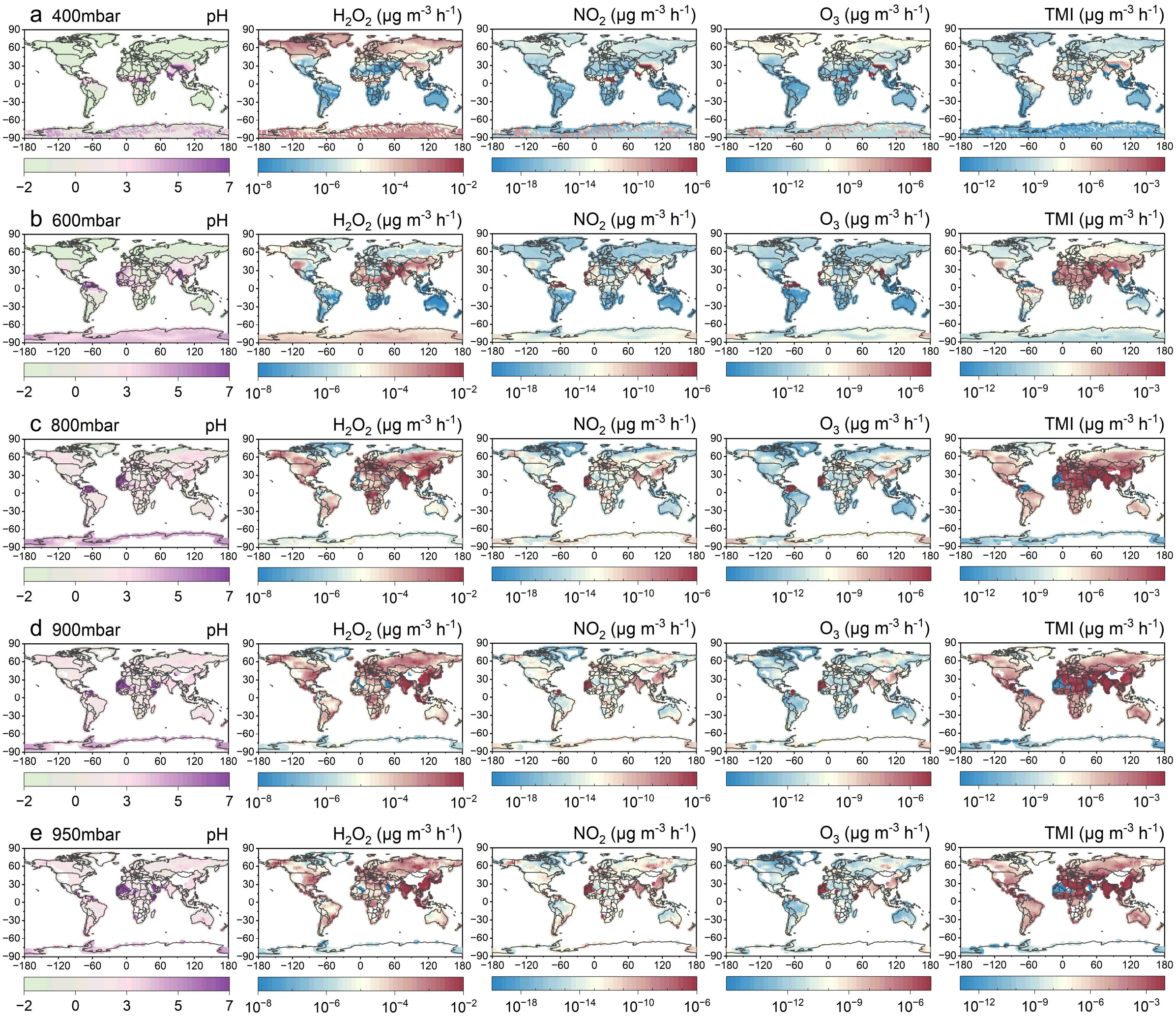

Fig. S10. Aerosol pH and aerosol sulfate formation rates through four aqueous oxidation pathways at different atmospheric pressure levels in July 2019. (a)-(e) indicated the results at 400 mbar, 600 mbar, 800 mbar, 900 mbar, and 950 mbar, respectively.

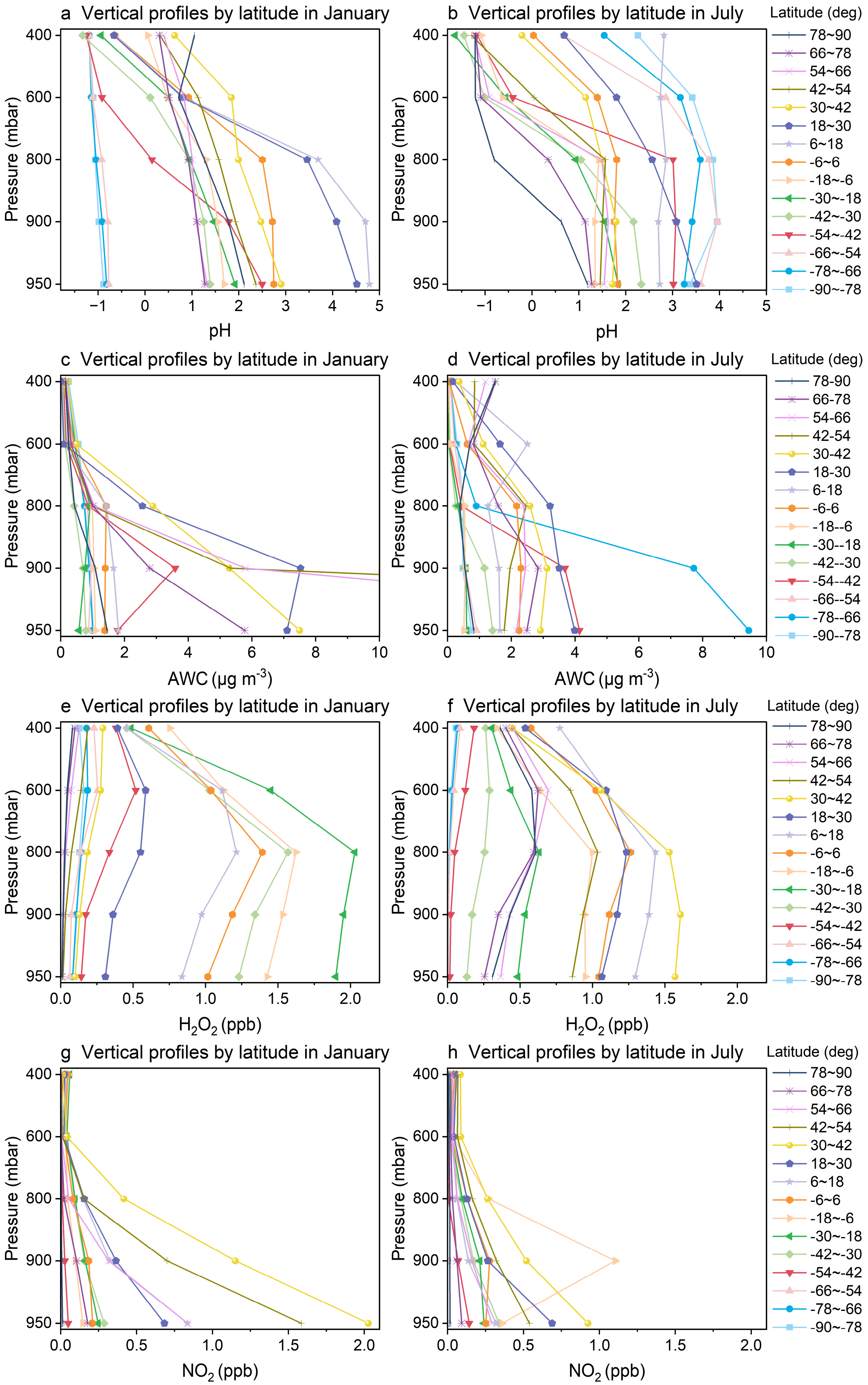

a Vertical profiles by latitude in January
b Vertical profiles by latitude in July
c Vertical profiles by latitude in January
d Vertical profiles by latitude in July
e Vertical profiles by latitude in January
f Vertical profiles by latitude in July
g Vertical profiles by latitude in January
h Vertical profiles by latitude in July
Pressure (mbar)
pH
AWC (μg m-3)
H2O2 (ppb)
NO2 (ppb)
Latitude (deg)
78~90
66~78
54~66
42~54
30~42
18~30
6~18
-6~6
-18~-6
-30~-18
-42~-30
-54~-42
-66~-54
-78~-66
-90~-78

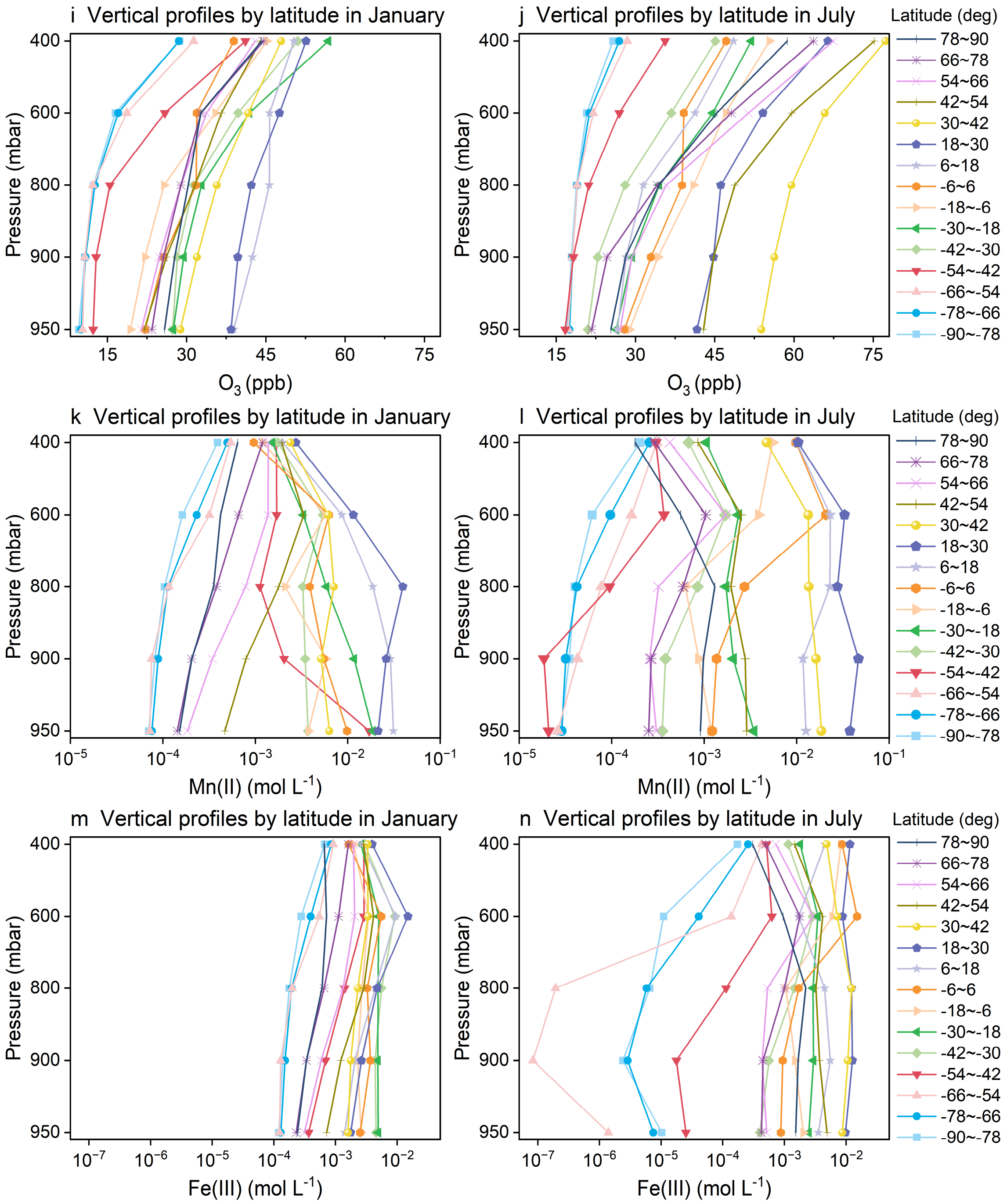

Fig. S11. Vertical distribution of aerosol pH, AWC, H_2O_2, NO_2, O_3, Mn(II), and Fe(III), averaged by latitude.

(a) aerosol pH, (c) AWC, (e) H_2O_2, (g) NO_2, (i) O_3, (k) Mn(II), and (m) Fe(III) indicated the corresponding values in January 2019. (b) aerosol pH, (d) AWC, (f) H_2O_2, (h) NO_2, (j) O_3, (l) Mn(II), and (n) Fe(III) indicated the corresponding values in July 2019. Obvious differences were shown between 950 mbar, 900 mbar, 800 mbar, 600 mbar, and 400 mbar pressure layers.

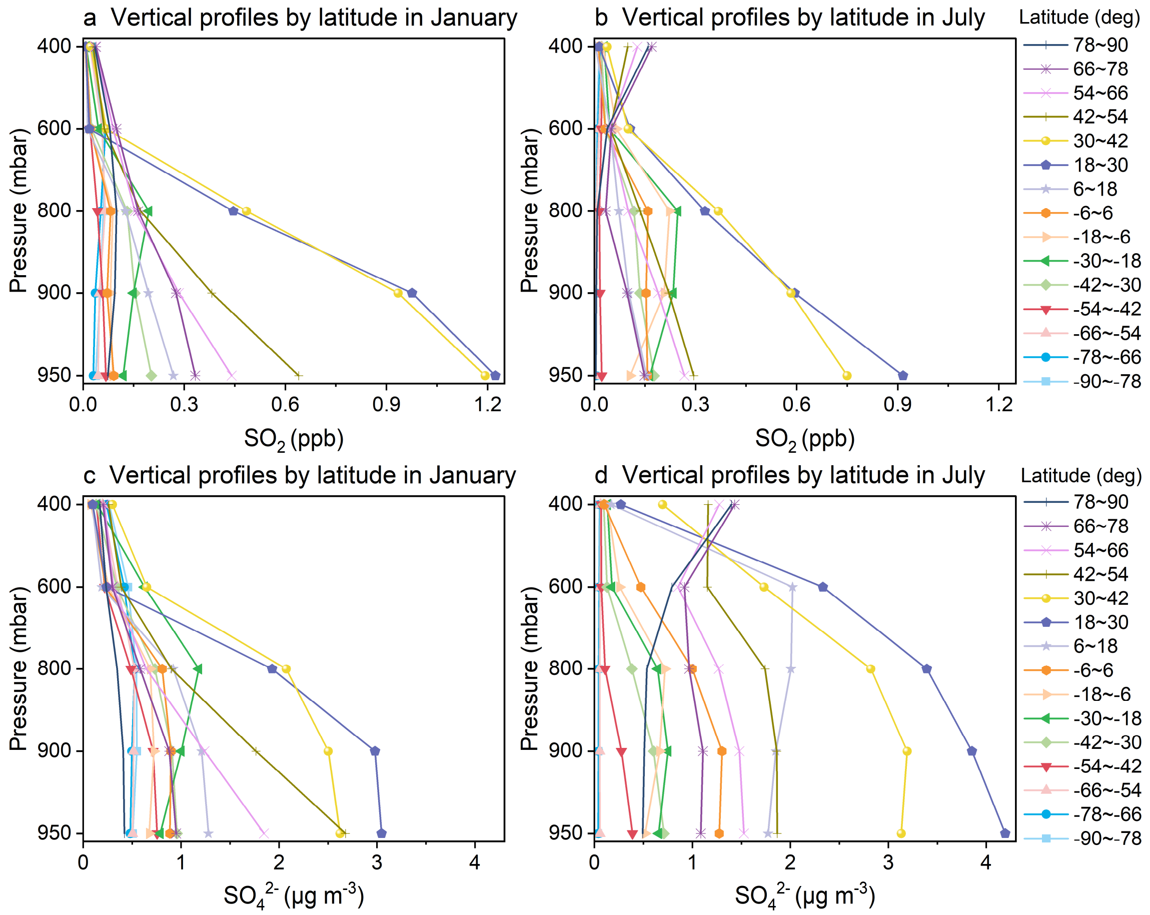

Fig. S12. Vertical distribution of SO_2 and SO_4^{2-} averaged by latitude.

(a) SO_2 and (c) SO_4^{2-} indicated the corresponding values in January 2019. (b) SO_2 and (d) SO_4^{2-} indicated the corresponding values in July 2019. Similar changes of SO_2 and SO_4^{2-} were shown in pressure layers of 950 mbar, 900 mbar, 800 mbar, 600 mbar, and 400 mbar.

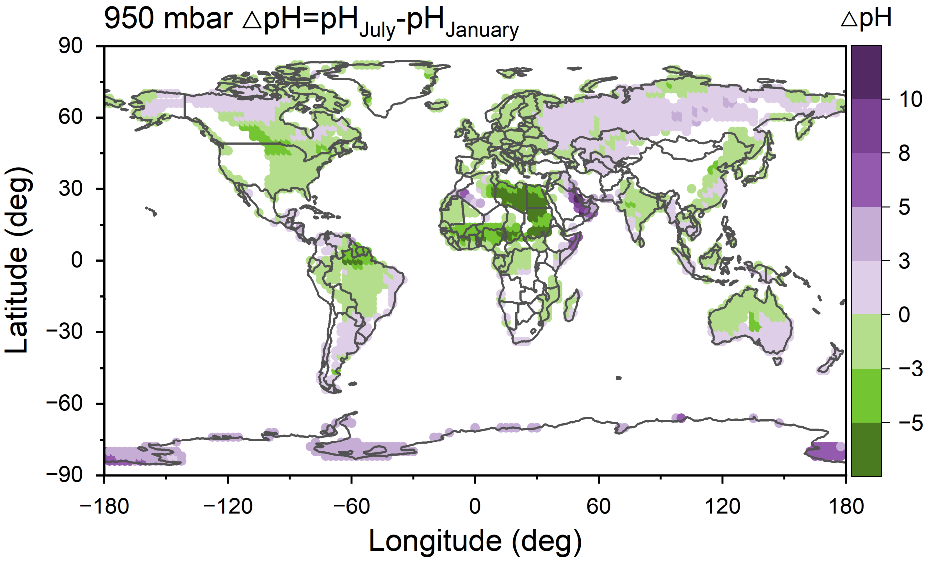

Fig. S13. The global difference in pH at 950 mbar between January and July 2019. △pH>0 indicated $pH_{July}>pH_{January}$. △pH<0 indicated $pH_{July}<pH_{January}$. The aerosol pH in most continental regions decreased in July, indicating the increase of aerosol acidity in July.

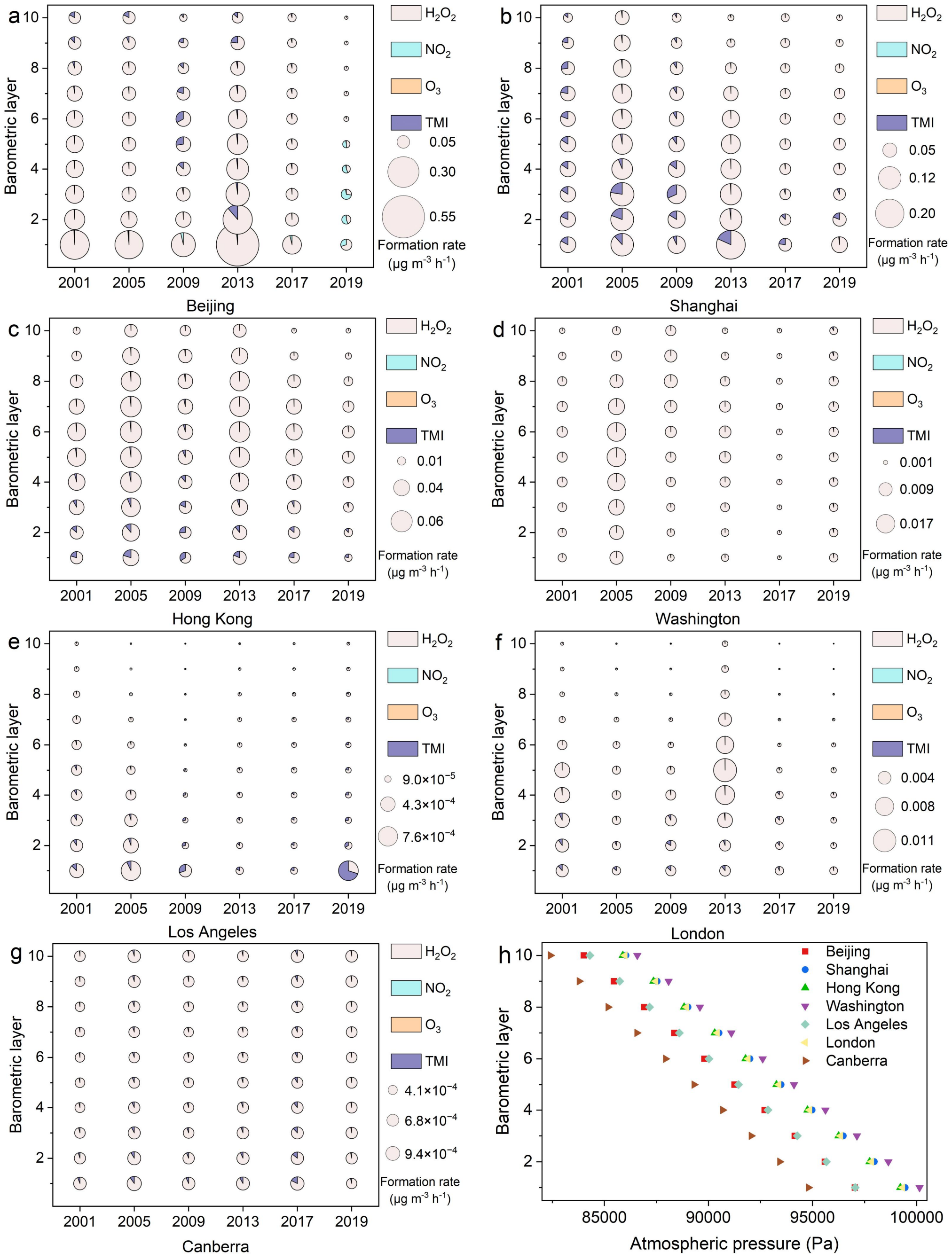

Fig. S14. Aerosol aqueous sulfate formation pathways in ten atmospheric layers near the surface of the typical urban areas.

(a) Beijing, (b) Shanghai, (c) Hong Kong, (d) Washington, (e) Los Angeles, (f) London, (g) Canberra, and (h) the atmospheric pressure in ten atmospheric layers near the surface of the typical urban areas. The H_2O_2 pathway showed the predominant role in most situations. NO_2 and TMI pathways also play critical parts in sulfate formation in some scenarios, like Beijing and Los Angeles, respectively.

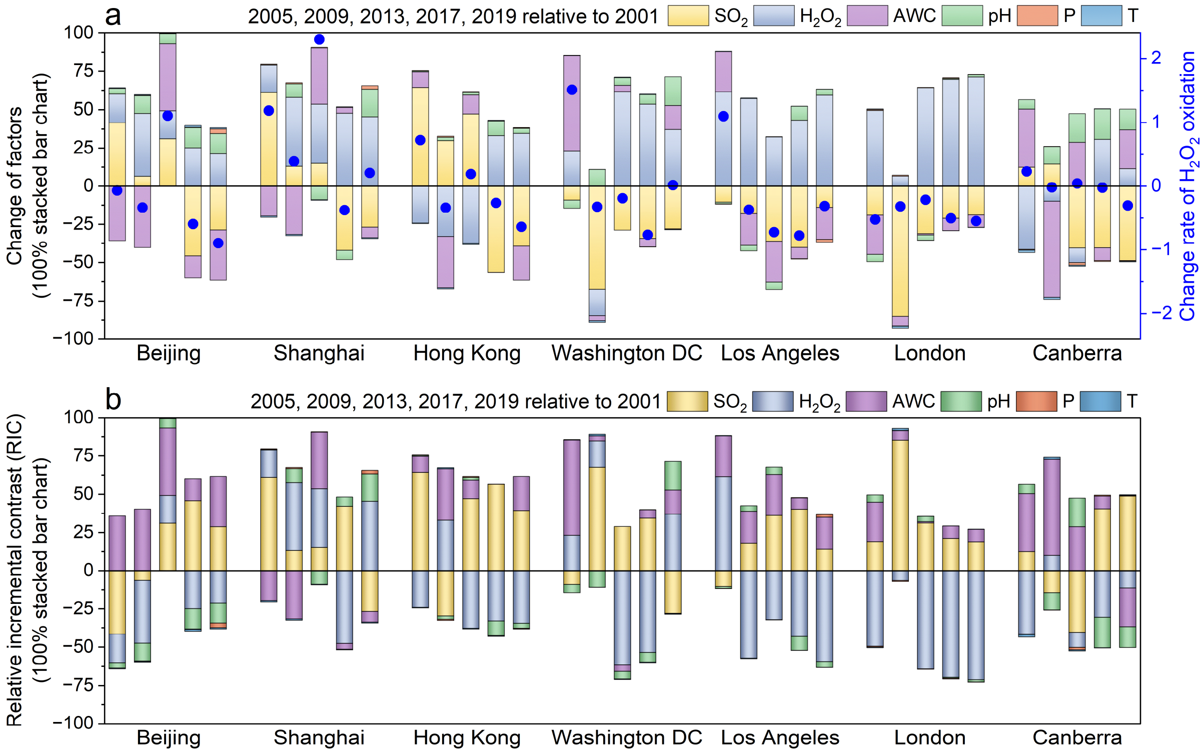

Fig. S15. Drivers of H_2O_2 pathway in typical urban areas in recent 20 years.

(a) Change tendency of H_2O_2 oxidation and the influence factors in 2005, 2009, 2013, 2017, and 2019, compared with that in 2001. (b) The relative incremental contrast (RIC), i.e., the change rate of each factor divided by the change rate of the H_2O_2 pathway, shown by 100% stacked bar charts.

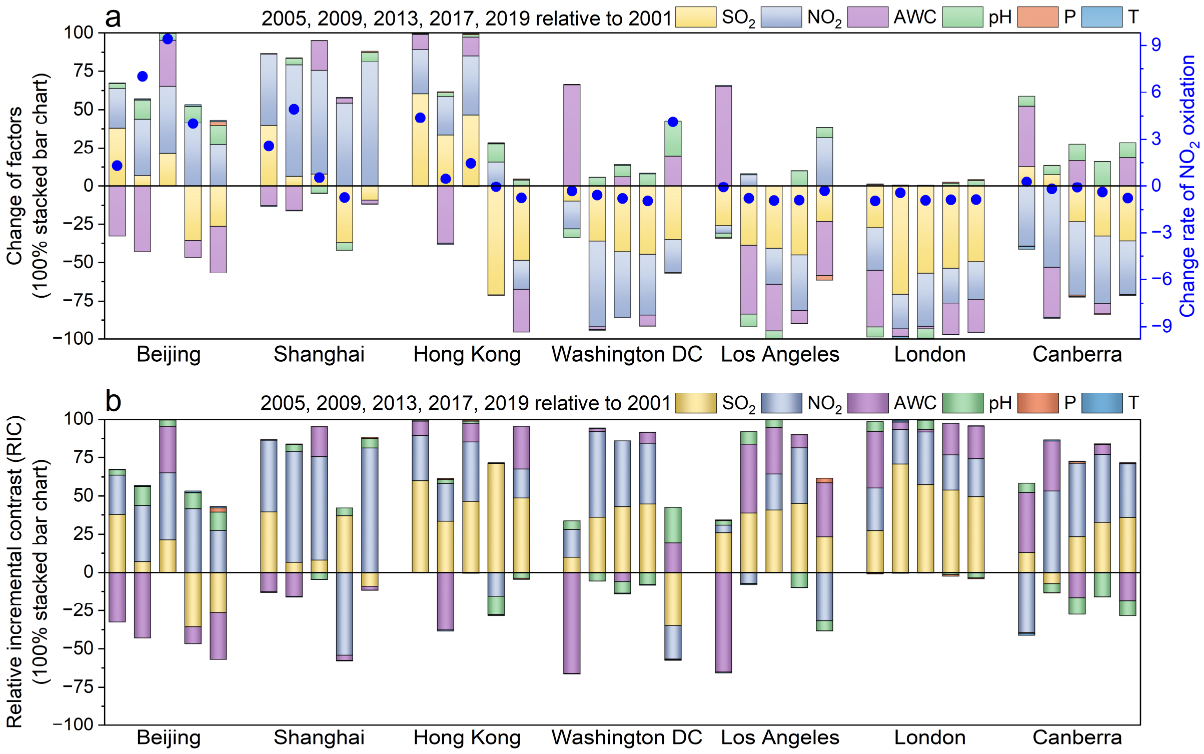

Fig. S16. Drivers of NO_2 pathway in typical urban areas in recent 20 years.

(a) Change tendency of NO_2 oxidation and the influence factors in 2005, 2009, 2013, 2017, and 2019, compared with that in 2001. (b) The relative incremental contrast (RIC), i.e., the change rate of each factor divided by the change rate of the NO_2 pathway, shown by 100% stacked bar charts.

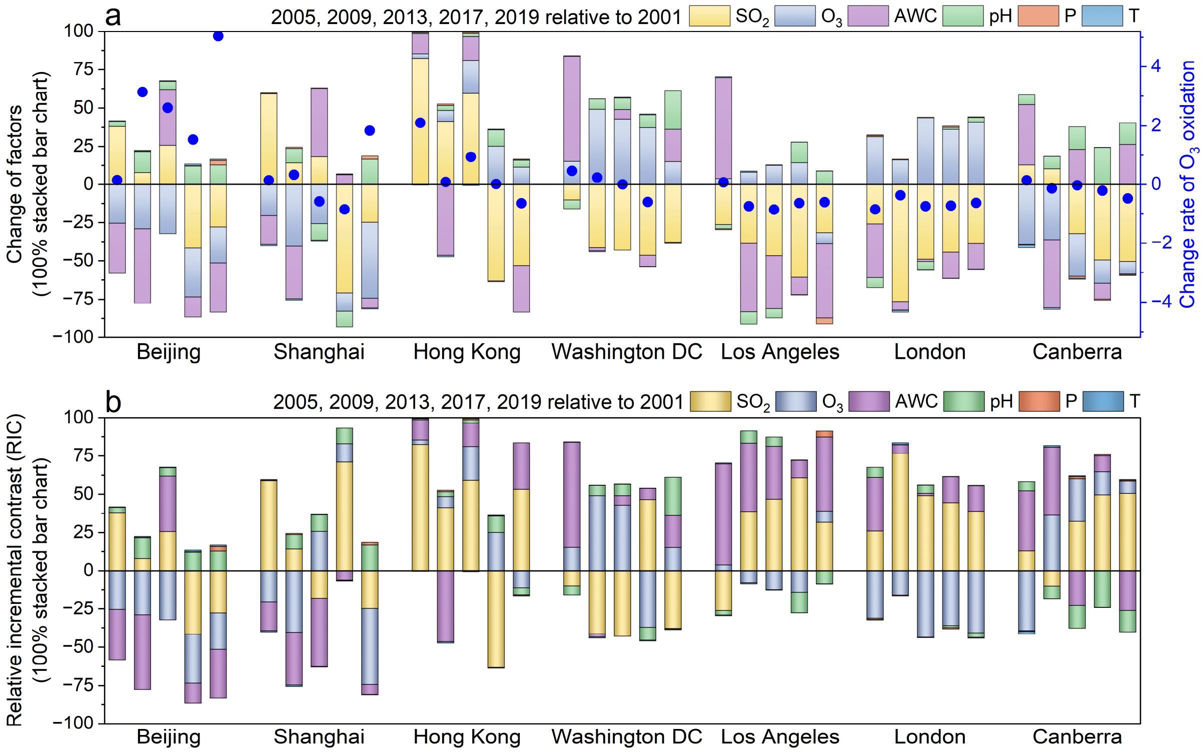

Fig. S17. Drivers of O_3 pathway in typical urban areas in recent 20 years.

(a) Change tendency of O_3 oxidation and the influence factors in 2005, 2009, 2013, 2017, and 2019, compared with that in 2001. (b) The relative incremental contrast (RIC), i.e., the change rate of each factor divided by the change rate of the O_3 pathway, shown by 100% stacked bar charts.

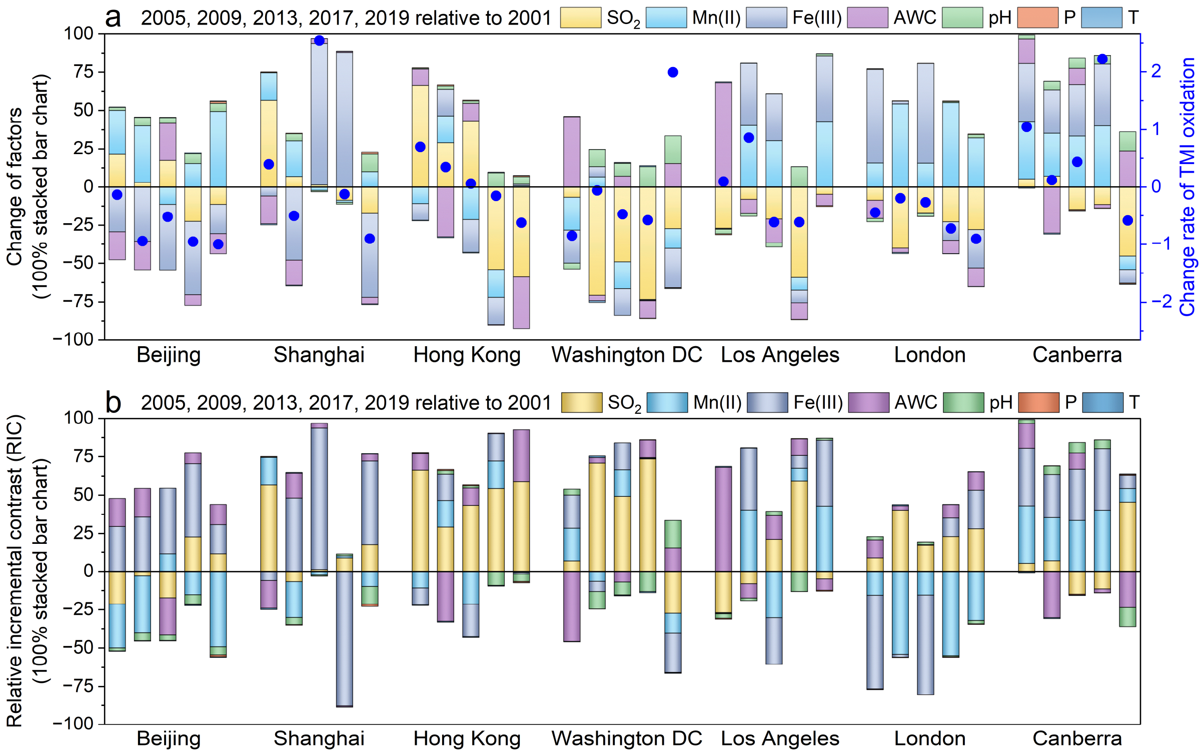

Fig. S18. Drivers of TMI pathway in typical urban areas in recent 20 years.

(a) Change tendency of TMI oxidation and the influence factors in 2005, 2009, 2013, 2017, and 2019, compared with that in 2001. (b) The relative incremental contrast (RIC), i.e., the change rate of each factor divided by the change rate of the TMI pathway, shown by 100% stacked bar charts.

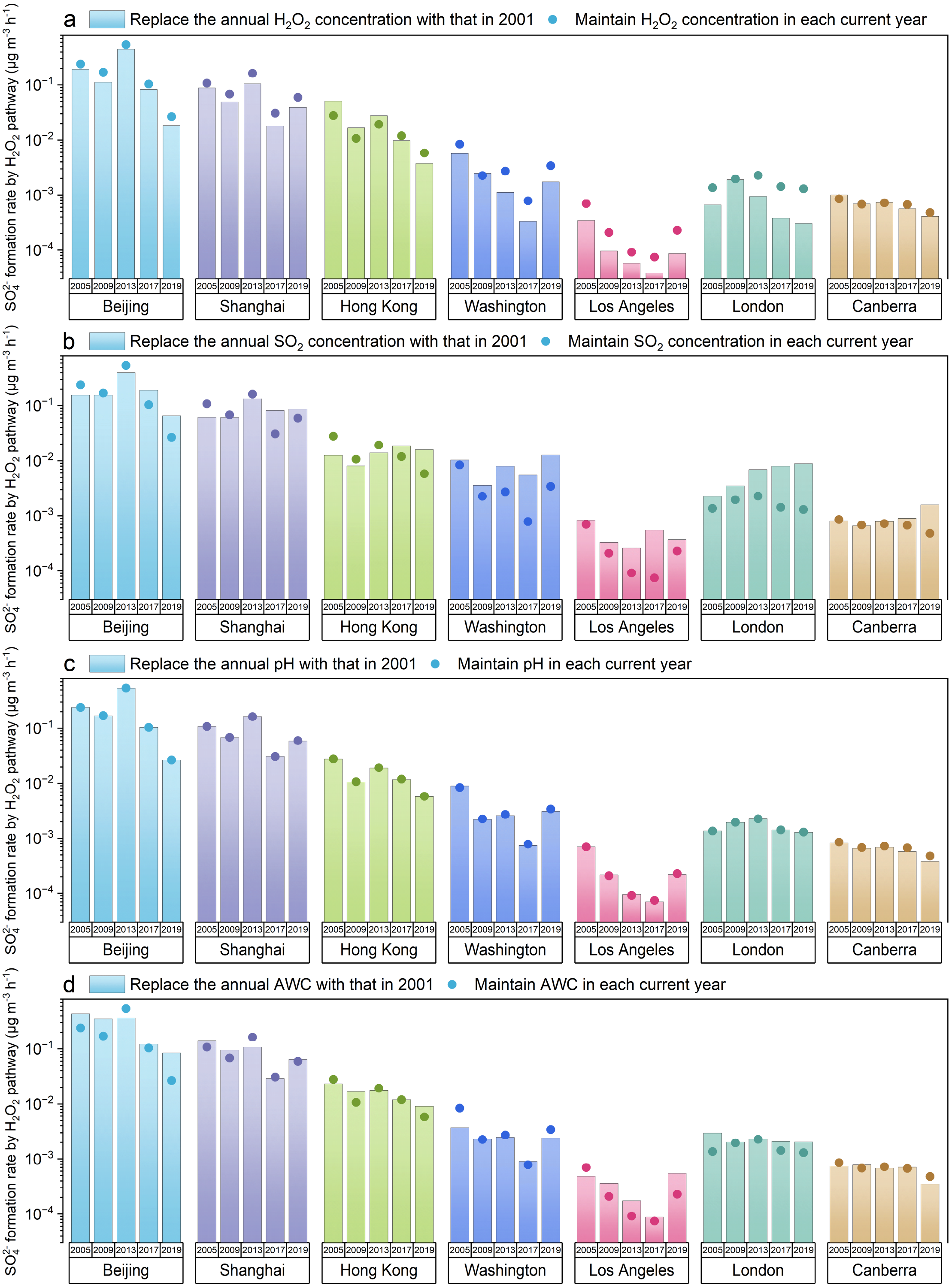

Fig. S19. Changes in aerosol sulfate production by the H_2O_2 pathway with fixation of H_2O_2, SO_2, pH, and AWC at 2001 levels.

The results are represented in (a)-(d), respectively. The dots are the current formation rates that maintain the conditions in each year. The columns are the changed formation rate after fixing the conditions in 2001.

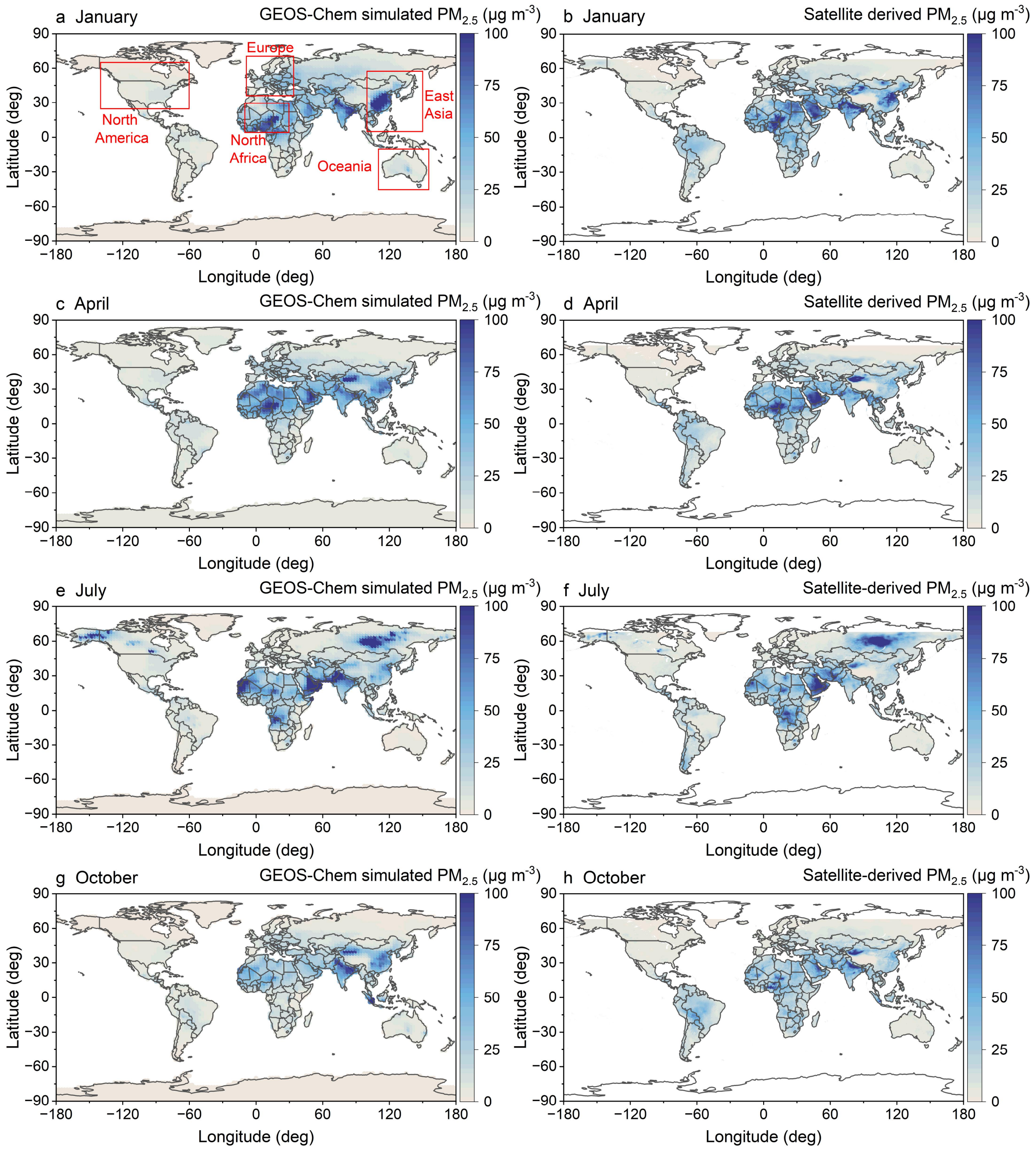

Fig. S20. Spatial distribution of $PM_{2.5}$ concentration from GEOS-Chem simulation and satellite-derived reanalysis dataset in January, April, July, and October 2019.

(a), (c), (e), and (g) GEOS-Chem simulated $PM_{2.5}$ in January, April, July, and October. (b), (d), (f), and (h) Satellite-derived $PM_{2.5}$ in January, April, July, and October. We utilized the reanalysis dataset "Satellite-derived $PM_{2.5}$" from the Atmospheric Composition Analysis Group at Washington University in St. Louis (https://sites.wustl.edu/acag/datasets/surface-pm2-5/, last access: 2024-3-31).

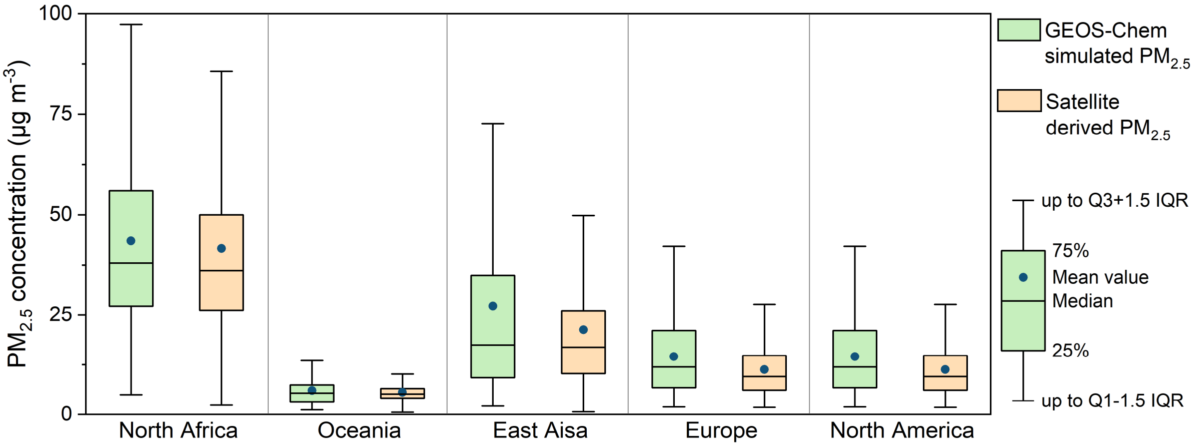

Fig. S21. Comparison of $PM_{2.5}$ concentration between GEOS-Chem simulation and satellite-derived reanalysis dataset in North Africa, Oceania, East Asia, Europe, and North America.

North Africa (5°N−30°N, 10°W−30°E), Oceania (10°S−45°S, 110°E−155°E), East Asia (5°N−58°N, 100°E−150°E), Europe (37°N−70°N, 9°W−34°E), and North America (25°N−65°N, 60°W−140°W) are focused for further comparison. The relevant datasets for reference and validation can be referred to https://doi.org/10.6084/m9.figshare.24967032.

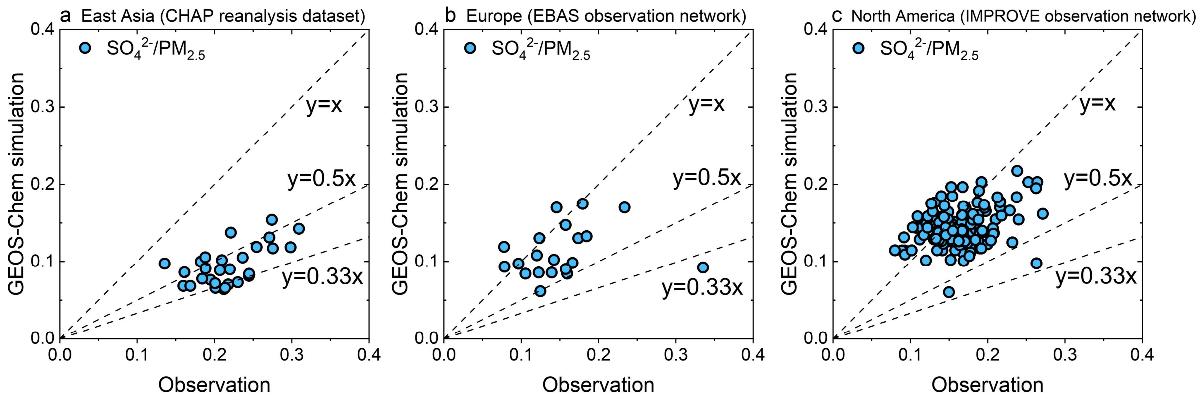

Fig. S22. $SO_4^{2-}/PM_{2.5}$ from GEOS-Chem simulation and observation in 2019.

(a) East Asia. (b) Europe. (c) North America. We compared the concentration ratio of $SO_4^{2-}/PM_{2.5}$ between GEOS-Chem model simulations and field observations/reanalysis datasets. We gathered the publicly available data from the CHAP reanalysis dataset in China (https://weijing-rs.github.io/product.html), the EBAS observation network in European countries (https://ebas-data.nilu.no/Default.aspx), and the IMPROVE observation network in the United States (https://views.cira.colostate.edu/fed/Express/ImproveData.aspx). Among them, a total of 20 points from EBAS and 142 points from IMPROVE were selected, along with 28 provincial capital cities in China.

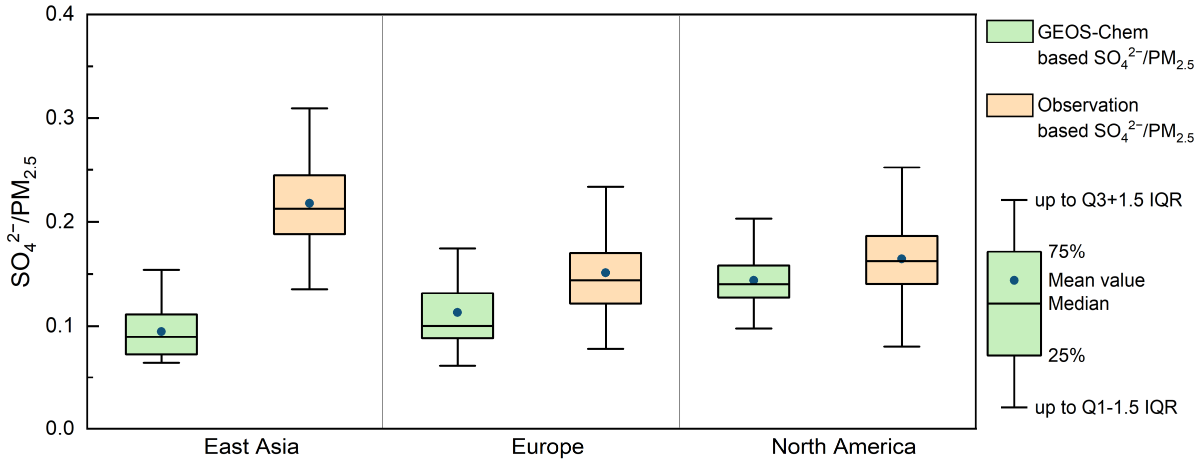

Fig. S23. Comparison of $SO_4^{2-}/PM_{2.5}$ between GEOS-Chem simulation and observation in East Asia, Europe, and North America.

The relevant datasets for reference and validation can be referred to https://doi.org/10.6084/m9.figshare.24967032.

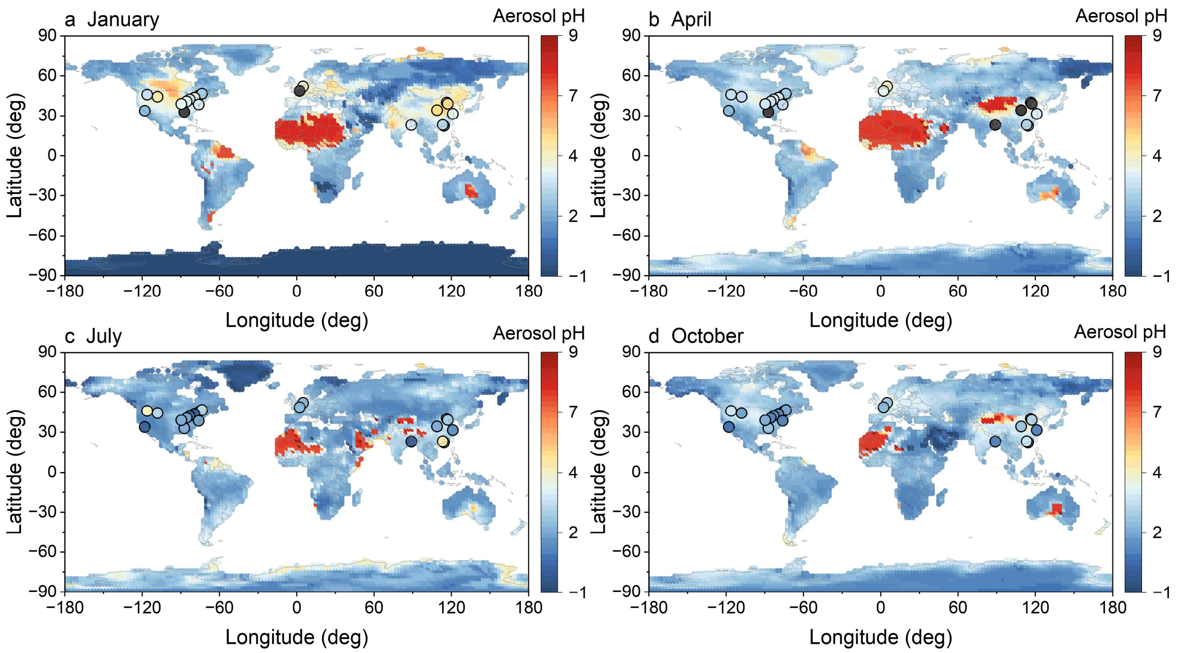

Fig. S24. Aerosol pH from GEOS-Chem simulation and observation-based estimation in January, April, July, and October.

(a) January, (b) April, (c) July, and (d) October. Aerosol pH from GEOS-Chem simulation in 2019 is represented as a spatial distribution map and observation-based estimation is represented as discrete points.

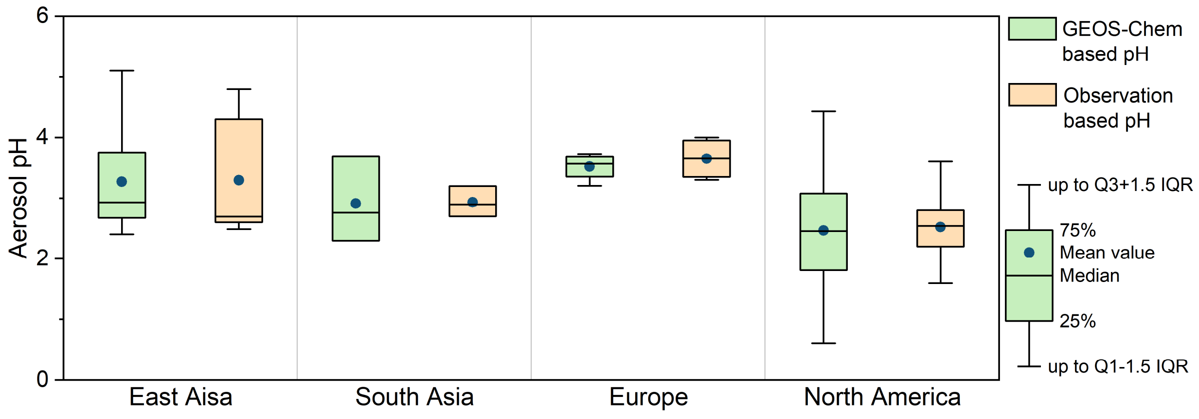

Fig. S25. Comparison of aerosol pH between GEOS-Chem simulation and observation-based estimation in East Asia, South Asia, Europe, and North America.

The relevant datasets for reference and validation can be referred to https://doi.org/10.6084/m9.figshare.24967032.

Supplementary References

1. Seinfeld, J. H. & Pandis, S. N. Atmospheric Chemistry and Physics: From Air Pollution to Climate Change. (2016).
2. Cheng, Y. *et al.* Reactive nitrogen chemistry in aerosol water as a source of sulfate during haze events in China. *Sci. Adv.* **2**, e1601530 (2016).
3. Liu, T. & Abbatt, J. P. D. Oxidation of sulfur dioxide by nitrogen dioxide accelerated at the interface of deliquesced aerosol particles. *Nat. Chem.* **13**, 1173–1177 (2021).
4. Ibusuki, T. & Takeuchi, K. Sulfur dioxide oxidation by oxygen catalyzed by mixtures of manganese(II) and iron(III) in aqueous solutions at environmental reaction conditions. *Atmos. Environ. (1967)* **21**, 1555–1560 (1987).
5. He, P. *et al.* Isotopic constraints on heterogeneous sulfate production in Beijing haze. *Atmos. Chem. Phys.* **18**, 5515–5528 (2018).
6. Liu, T., Chan, A. W. H. & Abbatt, J. P. D. Multiphase oxidation of sulfur dioxide in aerosol particles: Implications for sulfate formation in polluted environments. *Environ. Sci. Technol.* **55**, 4227–4242 (2021).
7. Ye, C. *et al.* A critical review of sulfate aerosol formation mechanisms during winter polluted periods. *J. Environ. Sci.* **123**, 387–399 (2023).
8. Gao, J. *et al.* Targeting atmospheric oxidants can better reduce sulfate aerosol in China: H_2O_2 aqueous oxidation pathway dominates sulfate formation in haze. *Environ. Sci. Technol.* **56**, 10608–10618 (2022).
9. Liu, T., Clegg, S. L. & Abbatt, J. P. D. Fast oxidation of sulfur dioxide by hydrogen peroxide in deliquesced aerosol particles. *Proc. Natl. Acad. Sci. U.S.A.* **117**, 1354–1359 (2020).
10. Su, H., Cheng, Y. & Pöschl, U. New multiphase chemical processes influencing atmospheric aerosols, air quality, and climate in the Anthropocene. *Acc. Chem. Res.* **53**, 2034–2043 (2020).
11. Yu, C. *et al.* Ionic strength enhances the multiphase oxidation rate of sulfur dioxide by ozone in aqueous aerosols: Implications for sulfate production in the marine atmosphere. *Environ. Sci. Technol.* **57**, 6609–6615 (2023).
12. Pye, H. O. T. *et al.* The acidity of atmospheric particles and clouds. *Atmos. Chem. Phys.* **20**, 4809–4888 (2020).
