# Supplementary material for: Hydrogen peroxide serves as pivotal fountainhead for aerosol aqueous sulfate formation from a global perspective
Source: Nat Commun. 2024 May 30;15:4625. doi: 10.1038/s41467-024-48793-1 (PMC11139875; doi:10.1038/s41467-024-48793-1)
Supplement: Supplementary file 3 — Reporting Summary [file 41467_2024_48793_MOESM3_ESM.pdf]

Corresponding author(s): Shaojie Song

Last updated by author(s): May 10, 2024

Reporting Summary

Nature Portfolio wishes to improve the reproducibility of the work that we publish. This form provides structure for consistency and transparency in reporting. For further information on Nature Portfolio policies, see our Editorial Policies and the Editorial Policy Checklist.

Statistics

For all statistical analyses, confirm that the following items are present in the figure legend, table legend, main text, or Methods section.

n/a	Confirmed	
☐	☒	The exact sample size (*n*) for each experimental group/condition, given as a discrete number and unit of measurement
☐	☒	A statement on whether measurements were taken from distinct samples or whether the same sample was measured repeatedly
☒	☐	The statistical test(s) used AND whether they are one- or two-sided<br>*Only common tests should be described solely by name; describe more complex techniques in the Methods section.*
☐	☒	A description of all covariates tested
☐	☒	A description of any assumptions or corrections, such as tests of normality and adjustment for multiple comparisons
☐	☒	A full description of the statistical parameters including central tendency (e.g. means) or other basic estimates (e.g. regression coefficient) AND variation (e.g. standard deviation) or associated estimates of uncertainty (e.g. confidence intervals)
☒	☐	For null hypothesis testing, the test statistic (e.g. *F*, *t*, *r*) with confidence intervals, effect sizes, degrees of freedom and *P* value noted<br>*Give P values as exact values whenever suitable.*
☒	☐	For Bayesian analysis, information on the choice of priors and Markov chain Monte Carlo settings
☐	☒	For hierarchical and complex designs, identification of the appropriate level for tests and full reporting of outcomes
☒	☐	Estimates of effect sizes (e.g. Cohen's *d*, Pearson's *r*), indicating how they were calculated

Our web collection on statistics for biologists contains articles on many of the points above.

Software and code

Policy information about availability of computer code

Data collection	GEOS-Chem chemical transport model 13.3.4 (https://github.com/geoschem). ISORROPIA II aerosol thermodynamics module (http://isorropia.epfl.ch).
Data analysis	Matlab R2021b, Excel 2016, and Origin 2024

For manuscripts utilizing custom algorithms or software that are central to the research but not yet described in published literature, software must be made available to editors and reviewers. We strongly encourage code deposition in a community repository (e.g. GitHub). See the Nature Portfolio guidelines for submitting code & software for further information.

Data

Policy information about availability of data

All manuscripts must include a data availability statement. This statement should provide the following information, where applicable:

- Accession codes, unique identifiers, or web links for publicly available datasets
- A description of any restrictions on data availability
- For clinical datasets or third party data, please ensure that the statement adheres to our policy

The authors declare that the main data supporting the findings of this study are available within the article, its Supplementary Information file, and the Source data. Source data are provided with this paper, and can be accessed online via the figshare DOI: https://doi.org/10.6084/m9.figshare.24967032. The global reanalysis dataset "Satellite-derived PM2.5" was obtained from the Atmospheric Composition Analysis Group at Washington University in St. Louis (https://sites.wustl.edu/

acag/datasets/surface-pm2-5/). The reanalysis datasets of sulfate and PM2.5 for the China region were obtained from the ChinaHighAirPollutants (CHAP) dataset (https://weijing-rs.github.io/product.html). The field observation datasets of sulfate and PM2.5 were collected from the EBAS observation network in European countries (https://ebas-data.nilu.no/Default.aspx) and the IMPROVE observation network in the United States (https://views.cira.colostate.edu/fed/Express/ImproveData.aspx). Data analysis and draw designs were conducted based on Matlab R2021b, Excel 2016, and Origin 2024. The maps within images are prepared using the built-in shapefiles in Origin software. We do not contain thrid-party images.

Research involving human participants, their data, or biological material

Policy information about studies with human participants or human data. See also policy information about sex, gender (identity/presentation), and sexual orientation and race, ethnicity and racism.

Reporting on sex and gender	Not applicable.
Reporting on race, ethnicity, or other socially relevant groupings	Not applicable.
Population characteristics	Not applicable.
Recruitment	Not applicable.
Ethics oversight	Not applicable.

Note that full information on the approval of the study protocol must also be provided in the manuscript.

Field-specific reporting

Please select the one below that is the best fit for your research. If you are not sure, read the appropriate sections before making your selection.

☐ Life sciences ☐ Behavioural & social sciences ☒ Ecological, evolutionary & environmental sciences

For a reference copy of the document with all sections, see nature.com/documents/nr-reporting-summary-flat.pdf

Ecological, evolutionary & environmental sciences study design

All studies must disclose on these points even when the disclosure is negative.

Study description	We quantify the potential contributions of the aerosol aqueous pathways to global sulfate production based on simulations from the GEOS-Chem model and subsequent theoretical calculations. We focus on the global spatiotemporal variabilities for January, April, July, and October 2019, both surface and vertical spatial scales. We also investigate the temporal trends of aqueous sulfate formation pathways and the corresponding influencing factors in typical cities over 2001-2019 based on the simulations in 2001, 2005, 2009, 2013, 2017, and 2019. H2O2 oxidation could significantly influence continental regions worldwide, both horizontally and vertically. Over the past two decades, shifts in the formation pathways within typical cities reveal an intriguing trend: despite significant reductions in SO2 emissions, the increased atmospheric oxidation capacity, like rising H2O2 level, has prevented a steady decline in SO42⁻ concentrations. We highlight the equal importance of managing both oxidants and precursors for effective sulfate control, and abating oxidant levels would facilitate the benefit of SO2 reduction and the positive feedback in sulfate mitigation.
Research sample	This research is based on the simulations from the GEOS-Chem model and theoretical calculations. We focus on the rate of sulfate formation and the factors that affect it, such as precursors (SO2), oxidants(H2O2, O2, O3, NO2, OH), meteorological factors (temperature, relative humidity), and chemical factors (aerosol pH). We divided the globe into 2.5° (longitude) × 2° (latitude) grids for simulation to get the above data. The vertical grid contains 47 pressure levels from the surface to the mesosphere.
Sampling strategy	There was no sampling involved in this study.
Data collection	We performed research and collected data by model simulation and theoretical calculation. We analyzed data using Matlab R2021b, Excel 2016, and Origin 2024.
Timing and spatial scale	We focus on the global spatiotemporal variabilities of sulfate formation for January, April, July, and October 2019, both surface and vertical spatial scales (up to 400 mbar). In addition, we modeled long-term trends in January from 2001 to 2017 at four-year intervals.
Data exclusions	No data were excluded.
Reproducibility	The simulations in this study were reproducible.
Randomization	Not applicable.
Blinding	Not applicable.

Did the study involve field work? ☐ Yes ☒ No

Reporting for specific materials, systems and methods

We require information from authors about some types of materials, experimental systems and methods used in many studies. Here, indicate whether each material, system or method listed is relevant to your study. If you are not sure if a list item applies to your research, read the appropriate section before selecting a response.

Materials & experimental systems

n/a	Involved in the study
☒	☐ Antibodies
☒	☐ Eukaryotic cell lines
☒	☐ Palaeontology and archaeology
☒	☐ Animals and other organisms
☒	☐ Clinical data
☒	☐ Dual use research of concern
☒	☐ Plants

Methods

n/a	Involved in the study
☒	☐ ChIP-seq
☒	☐ Flow cytometry
☒	☐ MRI-based neuroimaging

Plants

Seed stocks	Not applicable.
Novel plant genotypes	Not applicable.
Authentication	Not applicable.
